# Desaturation of the Sphingofungin Polyketide Tail Results in Increased Serine Palmitoyltransferase Inhibition

**DOI:** 10.1128/spectrum.01331-22

**Published:** 2022-09-19

**Authors:** Sandra Hoefgen, Alexander U. Bissell, Ying Huang, Fabio Gherlone, Luka Raguž, Christine Beemelmanns, Vito Valiante

**Affiliations:** a Biobricks of Microbial Natural Product Syntheses, Leibniz Institute for Natural Product Research and Infection Biology, Hans Knöll Institute (HKI), Jena, Germany; b Faculty of Biological Sciences, Friedrich Schiller University Jena, Jena, Germany; c Chemical Biology of Microbe-Host Interactions, Leibniz Institute for Natural Product Research and Infection Biology, Hans Knöll Institute (HKI), Jena, Germany; d Faculty of Chemistry and Earth Sciences, Friedrich Schiller University Jena, Jena, Germany; Universidade de Sao Paulo

**Keywords:** Aspergillus, CoA-ligase, sphingofungins, molybdate, serine palmitoyltransferase

## Abstract

Serine palmitoyltransferase catalyzes the first step of the sphingolipid biosynthesis. Recently, sphingolipid homeostasis has been connected to several human diseases, making serine palmitoyltransferases an interesting therapeutic target. Known and efficient serine palmitoyltransferase-inhibitors are sphingofungins, a group of natural products isolated from fungi. To further characterize newly isolated sphingofungins, we designed an easy to use colorimetric serine palmitoyltransferase activity assay using FadD, which can be performed in 96-well plates. Because sphingofungins exert antifungal activitiy as well, we compared the *in vitro* assay results with an *in vivo* growth assay using Saccharomyces cerevisiae. The reported experiments showed differences among the assayed sphingofungins, highlighting an increase of activity based on the saturation levels of the polyketide tail.

**IMPORTANCE** Targeting the cellular sphingolipid metabolism is often discussed as a potential approach to treat associated human diseases such as cancer and Alzheimer's disease. Alternatively, it is also a possible target for the development of antifungal compounds, which are direly needed. A central role is played by the serine palmitoyltransferase, which catalyzes the initial and rate limiting step of sphingolipid *de novo* synthesis and, as such, the development of inhibitory compounds for this enzyme is of interest. Our work here established an alternative approach for determining the activity of serine palmitoyltransferase adding another tool for the validation of its inhibition. We also determined the effect of different modifications to sphingofungins on their inhibitory activity against serine palmitoyltransferase, revealing important differences on said activity against enzymes of bacterial and fungal origin.

## INTRODUCTION

Serine palmitoyltransferase (SPT) is an essential enzyme in eukaryotes, which catalyzes the first step during sphingolipid (SL) biosynthesis, namely, the condensation of palmitoyl-coenzyme A (CoA) with serine to form 3-ketodihydrosphingosine. From this starting point, SLs are further differentiated, e.g., into glycosphingolipids or sphingomyelins, and associated with distinct cellular processes (1). Interestingly, some bacteria synthesize sphingolipids using a similar biosynthetic route to eukaryotes. However, while in eukaryotic cells SPT is a membrane-bound heterodimeric enzyme (1, 2), the prokaryotic SPT enzymes are soluble and homo-dimeric (3). Moreover, it has been shown that the general catalytic mechanism of eukaryotic and bacterial SPTs is the same, with only some differences in their kinetics (4).

SLs are integral parts of eukaryotic cell membranes and are further involved in different cellular functions such as cell signaling, cell apoptosis, and phagocytosis (5). Defects in the delicate balance of SL homeostasis are linked to several human diseases like diabetes, cancer, or Alzheimer′s disease (1, 5, 6). This link to debilitating diseases makes enzymes of the SL biosynthesis, including SPT, important drug targets, as interference with the biosynthetic process could be potentially used to adjust a misbalanced sphingolipid homeostasis (7–9).

While some synthetic inhibitors of SPT have been developed (10–12), many others have been isolated from fungi (13). These inhibitors are categorized in a larger group referred to as sphingolipid inhibitors (SIs). Among the SIs are specific inhibitors of SPT, such as myriocin, sphingofungins, and lipoxamycin, but also inhibitors of other sphingolipid biosynthetic enzymes, such as fumonisins, which inhibit the ceramide synthase (14).

Identification, characterization, and development of additional specific SPT inhibitors requires methodologies to determine their inhibitory activity. So far, *in vitro* assays using either purified bacterial SPT or enriched eukaryotic microsomes, harboring SPT in their membranes, were developed. Yet the SPT activity itself was mainly determined by quantifying the production of 3-ketodihydrosphingosine by mass spectrometry based methods (4, 12) or by the incorporation of radioactively labeled substrates (15). While being effective, these methods are laborious and require specialized equipment and materials. A more accessible spectrophotometric assay, which determines SPT activity by quantifying released CoA, has been developed (16), but the costs associated with the required CoA esterified substrate decreases the viability of this approach for high-throughput screening.

Recently, we elucidated the biosynthesis of the known SPT inhibitors sphingofungins B-D in Aspergillus fumigatus (17). During this process we isolated the novel sphingofungin derivatives B_2_ and C_2_. Additionally, through pathway rewiring with genes obtained from Paecilomyces variotii, we were able to produce sphingofungin derivatives named as sphingofungin C_3_ and C_4_. All of these sphingofungins are missing the C-14 hydroxyl group and instead harbor a Δ12 C = C bond. Furthermore, work on the chemical synthesis of sphingofungin C has recently increased the number of available derivatives in our hands (18). To test these potential SPT inhibitors we developed an easy to use colorimetric SPT activity assay that can be performed in 96-well plates and therefore allows high-throughput experiments.

With this assay, which includes two bacterial enzymes, we determined and compared the SPT inhibition activity of different isolated and synthetic sphingofungin derivatives. We could show an increased toxicity related to the C = C bond in the polyketide tail. To complement our findings, we additionally used a resazurin-based assay in yeast to verify the inhibitory effect of SIs on eukaryotic cells.

## RESULTS AND DISCUSSION

To determine the inhibition of SPT, we initially adapted a method reported by Raman et al. (16), and tested it with the known SPT inhibitors myriocin and sphingofungin B (1) (Fig. S1). This method is very easy to handle, but, in our hands, it has not produced any significant results. The trend of inhibition was quite clear, but due to the high variability of the measurements, it was not possible to discriminate between the different concentrations tested. Consequently, we decided to design an alternative colorimetric assay by coupling the activity of the long-chain-fatty-acid-CoA:ligase FadD from Escherichia coli to the SPT isolated from Sphingomonas paucimobilis (19, 20). SPT condenses serine with palmitoyl-CoA leading to the production of 3-ketodihydrosphingosine and the parallel release of CoA. CoA is then required by FadD for the esterification of palmitic acid to reform palmitoyl-CoA, which is then used by SPT.

**FIG 1:**
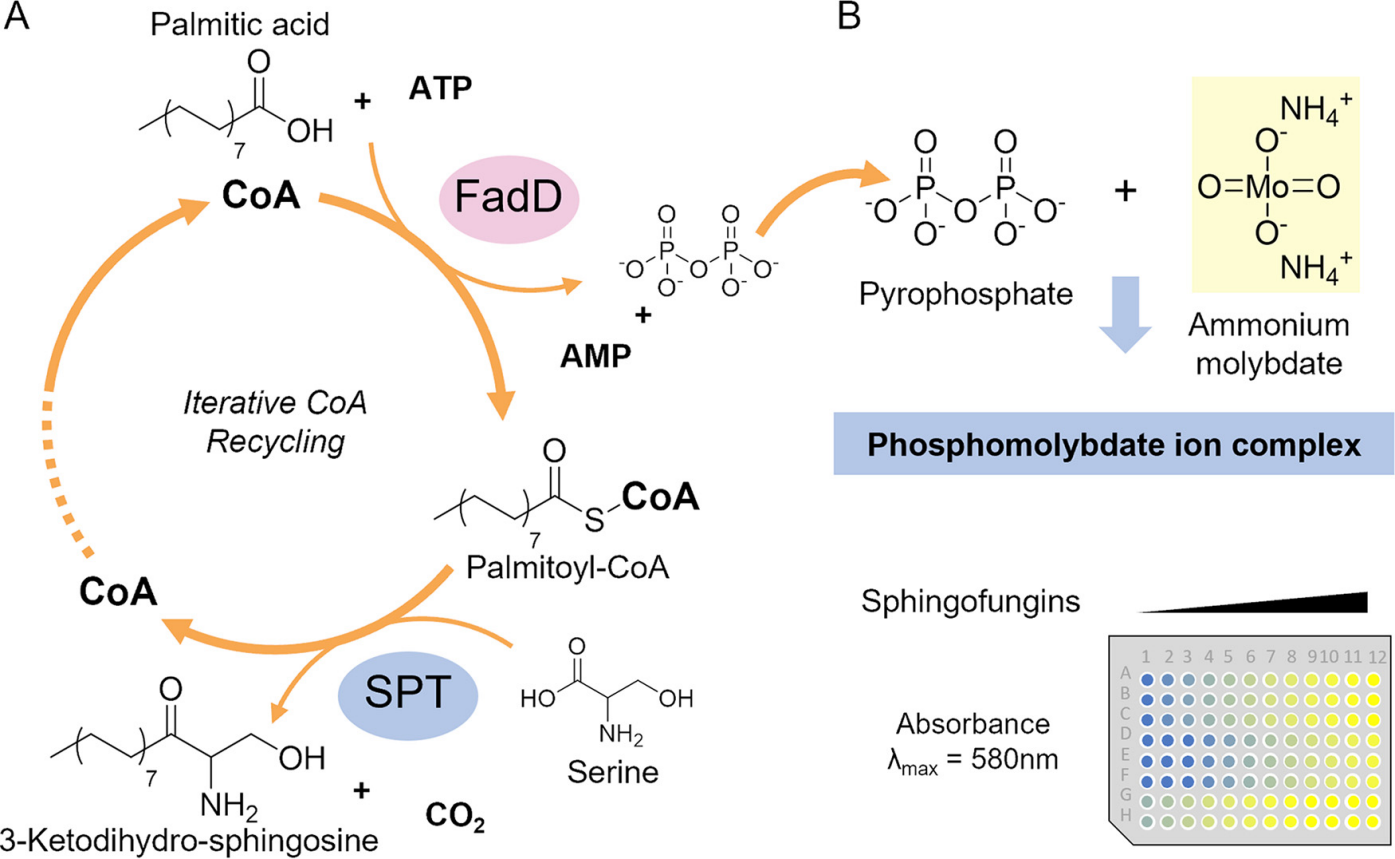
Principle of the SPT assay. (A) Visual representation of the reactions leading to the iterative CoA recycling and its resulting production of PPi. (B) The released PPi reacts with ammonium molybdate forming a bluish complex in reducing conditions. This complex has an absorbance at λmax = 580 and can be measured in 96-well plates.

FadD belongs to the family of adenylate forming enzymes, which produces an acyl-AMP intermediate prior to CoA esterification (21). Additionally, FadD presents a very large substrate promiscuity and is able to esterify a variety of fatty and phenolic acids (22). The activity of FadD is easily determined by quantifying the production of pyrophosphate (PPi) released through the consumption of ATP during the energy intensive esterification it catalyzes. Through limiting the availability of CoA, the activity of FadD is dependent on the iterative CoA recycling, which is driven by the SPT activity (Fig. 1A). Then, the use of an established molybdate assay for the quantification of PPi (23) enabled us to determine the overall SPT activity using a plate reader photometer (Fig. 1B).

**FIG 2:**
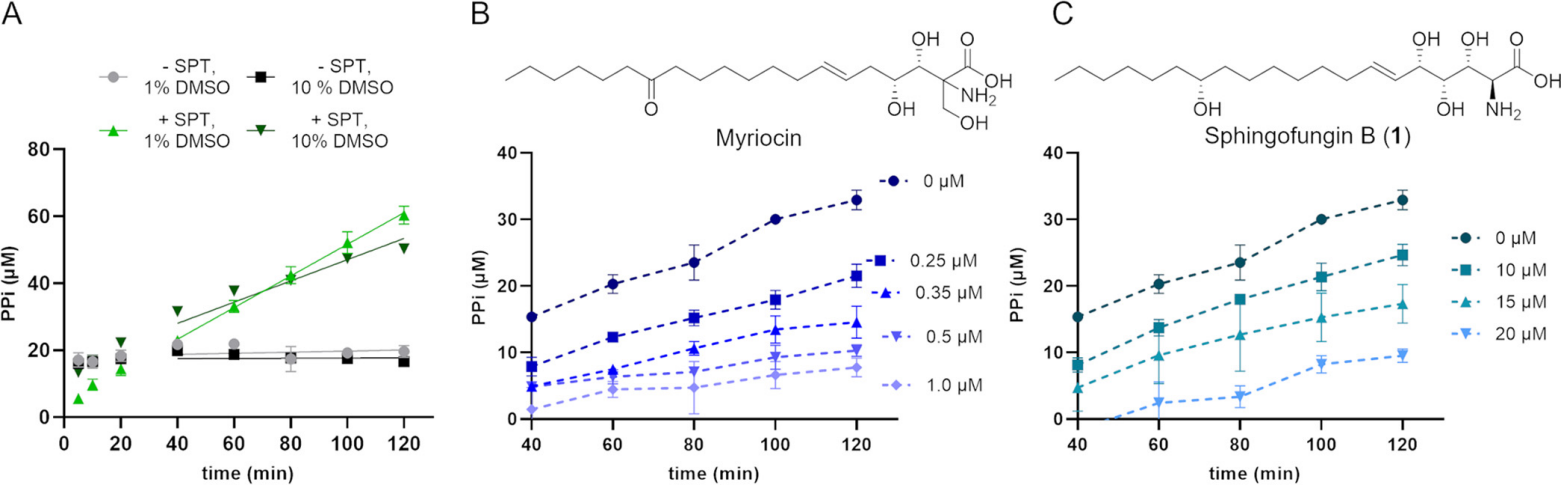
Initial set-up experiments of the SPT assay. SPT activity is shown as production of PPi over time. (A) SPT activity in the absence or presence of FadD and at different DMSO concentrations (1% and 10%). SPT activity in the presence of (B) myriocin and (C) sphingofungin B were used to validate the applicability of the assay. The sphingofungin concentration that shows medium SPT inhibition (15 μM) was then used for further experiments. All experiments were conducted in technical and biological triplicates. Error bars represent standard errors.

Initial experiments showed a linear increase of produced PPi in reactions containing the full iterative CoA cycle, whereas in reactions without SPT, and a disrupted CoA cycle, the PPi production was already quenched before the first measurements took place (Fig. 2A). Because many SIs are lipophilic, and require dimethylsulfoxide (DMSO) to be completely dissolved, we additionally tested its impact on the assay. We verified that a moderate concentration of the solvent does not significantly affect FadD activity, whereas the reduced linear climb in a SPT containing reaction with 10% DMSO compared with a reaction with 1% DMSO shows that the activity of SPT was influenced by the solvent. This was considered during all the experiments by adjusting control reactions with DMSO and keeping the solvent concentration equal among all individual samples. Moreover, we validated the influence of the concentration of serine on the assay activity and determined 5 mM to be the most optimal one (Fig. S2). Additionally, we verified the production of 3-ketodihydrosphingosine by our SPT reaction via high-performance liquid chromatography–high-resolution mass spectrometry (HPLC-HRMS) measurements (Fig. S3) and we further confirmed that neither myriocin, nor sphingofungins inhibit the activity of FadD (Fig. S4).

We first measured SPT inhibition using myriocin at different concentrations (24) as a control. As shown, the steady decrease of PPi production, and thus SPT activity, correlated with an increasing concentration of myriocin, thereby validating the assay (Fig. 2B). Next, we tested the inhibitory activity of sphingofungin B (1) at different concentrations. As with myriocin, the PPi production decreased correlating with the increasing concentration of 1, although much higher concentrations of 1 were required to achieve an inhibition comparable to that of myriocin (Fig. 2C). Overall, this confirmed differences of the activity between myriocin and 1 as already documented (10, 25).

**FIG 3:**
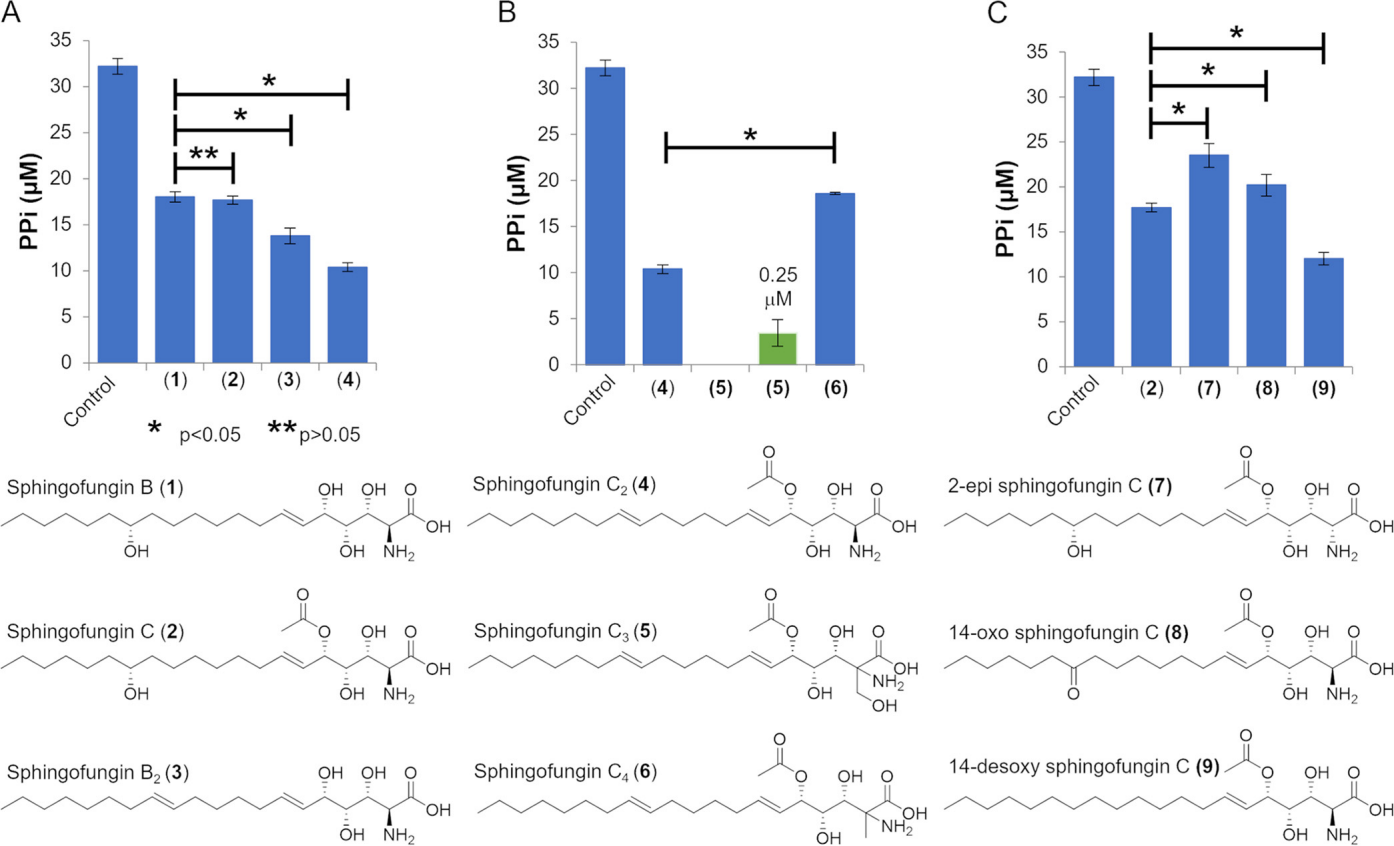
Comparative SPT inhibition assays. SPT activity is shown as production of PPi over time. (A) Comparison of SPT inhibitory activity of the different sphingofungin derivatives B (1), C (2), B_2_ (3) and C_2_ (4), together with a control reaction without any addition of an inhibi-tor. (B) Comparison of SPT inhibitory activity of sphingofungin C2 (4) and the sphingofungin derivatives C_3_ (5) and C4 (6), together with a control reaction without any addition of an inhibitor. The green column indicates the result of an assay performed with 0.25 μM inhibitor instead of 15 μM. Results with 15 μM 5 are shown as zero. (C) Comparative assay with sphingofungin C (2) and chemically synthesized sphingofungin derivatives, 2-epi sphingo-fungin C (5), 14-oxo sphingofungin C (6); and 14-desoxy sphingofungin C (7), together with a control reaction. All experiments were conducted in technical and biological triplicates. Error bars show standard errors and statistical significance was determined using a one tailed, paired *t* test.

We then proceeded to compare the inhibitory activity of sphingofungin B (1) with sphingofungin C (2) and the novel sphingofungin derivatives B_2_ (3) and C_2_ (4) (17) (Fig. 3A). The results showed that 1 and 2 inhibit the production of PPi on an equal level. It is interesting to note that the sphingofungin derivatives 3 and 4 show a significantly stronger inhibition of PPi production compared with 1 and 2, with 4 being the strongest inhibitor of the four. This would conclude that either, or both the missing C-14 hydroxylation and the additional Δ12 C = C bond are increasing the inhibitory activity of the two compounds. Contrary to this observation, Kobayashi et al. (26) reported that the C-14 hydroxylation is important to the inhibition of SPT by sphingofungins. However, in the reported experiments, the authors used the eukaryotic SPT enriched in membrane fractions from the mammalian CHO-K1 cells; moreover, the sphingofungin derivative tested missing the C-14 hydroxylation did not contain the Δ12 desaturation (26).

Sphingofungins C_3_ (5) and C_4_ (6) were detected in extracts obtained from the previously created strain A. fumigatus *xyl-G sphAPv* (17) via HPLC-HRMS and tandem high-resolution mass spectrometry (HRMS^2^) analyses (Fig. S5). After upscaling, larger quantities of these compounds were purified, elucidated by nuclear magnetic resonance spectroscopy (NMR) (Table S1 and S2; Note S1 and S2), and tested here. These compounds are of interest due to their headgroup which is similar to myriocin.

As shown, the inhibition of SPT by 6 was significantly lower compared with sphingofungin C_2_ (4), but on a similar level to sphingofungin C (2) (Fig. 3B). This shows that the methyl group has a negative impact on the inhibition against the bacterial SPT, as previously indicated by the comparison of inhibition by sphingofungin B and sphingofungin F (27). Indeed, compound 5, with a hydroxymethylated C1, showed an increased activity. For this compound, the activity was not determinable using a concentration of 15 μM, as the measured PPi was below the FadD base activity; thus, it was lowered to 0.25 μM, still resulting in an almost complete inhibition of SPT. The increase of activity of 5 compared with 4 was expected as it was already determined that the hydroxymethylation found in myriocin is an important functional group for its inhibitory mechanism against the bacterial SPT (28). Nonetheless, 5 appears to be even more potent than myriocin.

To complement our findings, we additionally tested and compared the recently chemically synthesized sphingofungin C derivatives 2-epi sphingofungin C (7), 14-oxo sphingofungin C (8) and 14-desoxy sphingofungin C (9, 18) (Fig. 3C). Also, 7 and 8 showed significantly reduced inhibition of PPi production, whereas with 7, significantly more PPi was produced than with 8. Interestingly, 9 showed a significant increase of inhibitory activity with an PPi production on a similar level as with 3 and 4. This indicates that the missing C-14 hydroxylation is the main cause for the increase of inhibitory activity of these three compounds, whereas the difference between 4 and 9 suggests a minor improvement introduced by the Δ12 desaturation.

**FIG 4:**
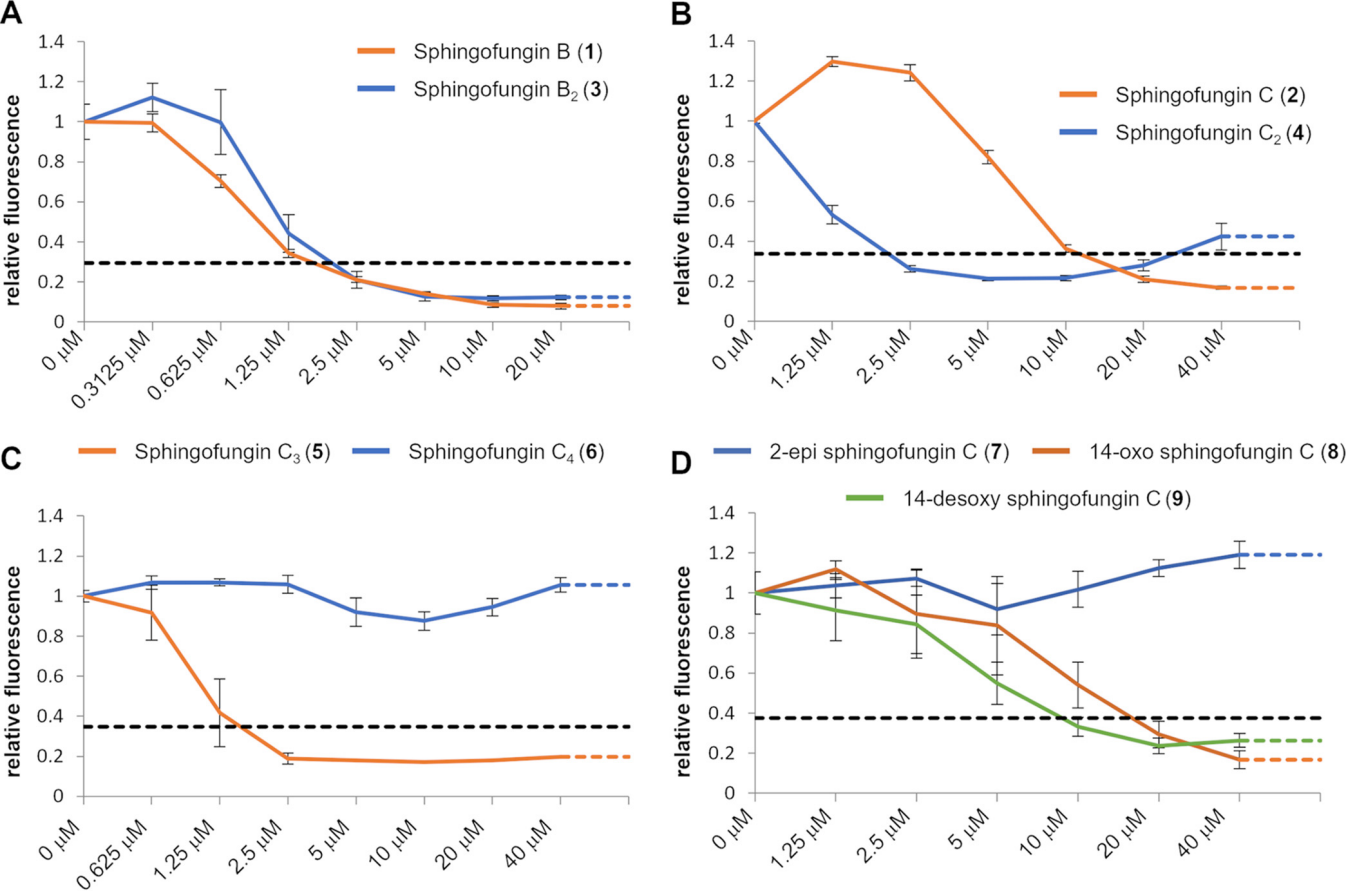
*In vivo* yeast resazurin assays. Metabolic activity is represented as relative fluorescence (*y* axis) depending on the concentration of the used inhibitor (*x* axis). The black dotted line indicates the relative fluorescence observed at an inhibiting concentration of hygromycin B and functions as the threshold at which S. cerevisiae is considered fully inhibited. The assay was performed comparing the inhibitory activity of isolated sphingofungin derivatives (A) sphingofungin B (1) and B_2_ (3); (B) sphingofungin C (2) and C_2_ (4); (C) sphingofungin C_3_ (5) and C_4_ (6); (D) the synthetic derivatives 2-epi sphingofungin C (7), 14-oxo sphingofungin C (8), and 14-desoxy sphingofungin C (9). All experiments were conducted in technical and biological triplicates. Error bars show the calculated standard errors. The calculated IC_50_ values of the here used compounds are reported in Table S3.

To further validate our data, we performed a resazurin assay with Saccharomyces cerevisiae (29, 30). The initial set-up of the assay was performed and validated by using different concentrations of myriocin (Fig. S6). We have then tested different concentrations of the isolated compounds. Inhibition was determined by comparison of the relative fluorescence with a positive control, incubation with the antifungal drug hygromycin B (31), and a negative control, absence of inhibitors. Both sphingofungin B (1) and B_2_ (3) fully inhibited S. cerevisiae growth at a concentration of 2.5 μM, with metabolic activity already decreased at 1.25 μM (Fig. 4A). Interestingly, the higher polyketide-desaturation present in sphingofungin B_2_ appeared not to be relevant here. Oppositely, sphingofungin C (2) and C_2_ (4) showed a significantly different activity in the *in vivo* assay (Fig. 4B), with 4 already inhibiting cell-growth at concentrations of 2.5 μM, whereas 2 requires a concentration of 10 μM. As such it is interesting that 4, which is acetylated, has a higher inhibitory activity than 2; this suggests a positive influence of the Δ12 C = C bond on antifungal activity of acetylated sphingofungins.

Concerning the additionally tested compounds, sphingofungin C_4_ (6) showed no inhibition of S. cerevisiae, further cementing that the methylation in the head group has a negative impact on the activity (Fig. 4C) (27), while sphingofungin C_3_ (5) was active as well at a concentration of 2.5 μM (Fig. 4B), with no observable increase to activity introduced by the C-1 hydroxymethylation.

Finally, we also tested the chemically synthesized sphingofungin derivatives *in vivo* (Fig. 4D). As previously proposed, the stereochemistry of the primary structure (C1 to C4) of sphingofungins is important to their inhibitory activity (18, 26). We could confirm that 2-epi sphingofungin C (7) showed no inhibition at the tested conditions, and that 14-oxo sphingofungin C (8) and 14-desoxy sphingofungin C (9) both show an inhibition of growth at 20 μM and 10 μM, respectively, in line with the *in vitro* SPT-assay.

Taken together, the *in vivo* tests confirmed that the increased inhibitory activity against S. cerevisiae observed by 4, compared with 2, is connected to the Δ12 desaturation, rather than the missing C-14 hydroxylation. Yet the absent C-14 hydroxylation does not negatively impact the inhibition of S. cerevisiae, as observed by comparing the synthetic compounds 14-oxo sphingofungin C (8) and 14-desoxy sphingofungin C (9).

### Concluding remarks.

We successfully established and validated a new approach for determining SPT activity by coupling the synthesis of 3-ketodihydrosphingosine and the accompanying release of CoA to the quantifiable release of PPi by FadD through the ATP dependent esterification of palmitic acid with aforementioned CoA. We applied this approach to determine and compare the SPT inhibitory activity of novel sphingofungin derivatives (17, 18) on the bacterial SPT of *S. paucimobilis* (19), which was used as a model to investigate the enzymatic mechanisms of SPT inhibition (10, 28). The comparison of our *in vitro* data, gained using the bacterial SPT with a simple *in vivo* assay, the antifungal activity against S. cerevisiae, revealed a striking difference in the inhibitory activity of the investigated sphingofungins, likely due to the structural differences between the SPTs of these organisms. With the reported assay we could confirm the importance of the hydroxymethylated C-1 in augmenting sphingofungin activity against the bacterial SPT, an effect not observed against the fungal SPT. Additionally, we discovered that the removal of the C-14 hydroxylation has a positive effect on the activity against the bacterial SPT, whereas it has no effect on the activity against the fungal SPT. We also highlighted that the Δ12 desaturation increases antifungal activity of C-5 *O*-acetylation sphingofungins and we have pointed out the importance of the polyketide desaturation-dependent toxicity, which has been overlooked so far.

## MATERIALS AND METHODS

### General cultivation and media.

In this study, strains from E. coli DH5α, E. coli BL21(DE3), and S. cerevisiae *BY4741* (Euroscarf) were used. E. coli cells were cultivated in LB media supplemented with appropriate selection markers and grown at 37°C either in liquid culture at 180 rpm or on plates, unless stated otherwise. S. cerevisiae cells were cultivated in yeast extract peptone dextrose (YPD) or synthetic dextrose (SD) media at 30°C in liquid culture at 180 rpm or on plates, unless stated otherwise.

### General molecular methods.

For amplification of desired DNA fragments from template DNA, the Phusion Flash High-Fidelity PCR Master Mix (Thermo Fisher Scientific) was used according to the conditions recommended by the supplier, unless stated otherwise. All primers used in this study are reported in Table S4. For DNA enzymatic digestions, restriction enzymes by New England Biolabs were used according to the supplier’s recommendations. DNA extraction from agarose gel was performed using the GeneJET Gel Extraction Kit (Thermo Fisher Scientific) following the protocol provided with the kit.

Seamless assembly of plasmid DNA from DNA fragments was performed using the NEBuilder Hifi DNA Assembly Kit (New England Biolabs) following the supplier’s instructions. Chemo competent E. coli cells prepared via the Mix & Go E. coli Transformation Kit and Buffer set (Zymo Research) were used for transformation with appropriate plasmid DNA or reaction mix. The transformation was performed following the kit’s instructions and selection was performed with appropriate selection markers.

Plasmid DNA purification was performed utilizing the NucleoSpin Plasmid minikit (Macherey-Nagel) following the instructions provided in the kit. All plasmids assembled in this study are reported in Table S5.

### Sequencing.

Sequencing of DNA was performed using the Ready 2 Run service from LGC genomics (Berlin).

### HPLC-HRMS analysis.

HPLC-HRMS analysis was performed using an LC-MS system consisting of a Q-Exactive Plus Hybrid Quadrupole Orbitrap mass spectrometer using electrospray ionization and a Dionex UltiMate 3000 UHPLC system (Thermo Fisher Scientific). Sample separation via HPLC was performed with a Kinetex C18 column (2.1 × 150 mm, 2.5 μm, 100 Å, Phenomenex) at a flowrate of 0.3 mL/min. For all samples, an injection volume of 3 μL was used with the following gradient elution of solvents A (water, 0.1% [vol/vol] formic acid) and B (acetonitrile, 0.1% [vol/vol] formic acid): 5% B for 5 min; a linear gradient to 98% B for 11.5 min, then 97% B for 3 min, a gradient to 5% B for 3 min. HRMS data were analyzed using the XCalibur software (Thermo Fisher Scientific).

### Purchased chemicals.

Reagents and solvents used in this study where purchased from Applichem, Thermo Fisher Scientific, Invivogen Europe, LI-COR GmbH, Life Technologies GmbH, Carl Roth, Sigma-Aldrich, Th. Geyer and VWR. They were used without further purification.

### Sphingofungin derivatives previously isolated from Aspergillus fumigatus.

Sphingofungin B (1), C (2), B_2_ (3), and C_2_ (4) were acquired from stocks isolated in our previous work. For strains, method of purification, and NMR analyses, see Bissell et al. (17).

### Purification of extracts for detection, isolation, and characterization of sphingofungins C_3_ and C_4_.

A. fumigatus *xyl-G sphA^Pv^* (Pv, Paecilomyces variotii) (strain described in Bissell et al. [17]) was inoculated in a preculture with Darken media (32) with 5 × 10^6^ spores/mL, and incubated at 37°C and 180 rpm overnight. Then, 2 mL of this culture was used to inoculate 20 mL V8-based production medium (167 mL/L V8-vegetable juice; 2.2 g/L trisodium citrate dihydrate; adjusted to pH 7.0 with sodium hydroxide, 20 g/L xylose). Cultures were incubated at 28°C for 6 days under static conditions. Extraction of cultures was performed by adding MeOH to a final concentration of 33% (vol/vol) and followed by homogenization using an Ultra turrax T18 digital (IKA). Homogenized cultures were incubated at room temperature for 1 h and then filtered through MN 615 filter papers (Macherey-Nagel). Crude extracts were prepurified using 200 mg Chromabond C18ec Silica Gel columns (Macherey-Nagel). Columns were conditioned using 2 mL 100% MeOH and 2 mL water, after which the crude extract was applied to the column. The columns were washed with water and 50% MeOH. Elution of compounds was achieved in 100% MeOH and evaporated using a Genevac EZ-2 sample concentrator (SP Scientific). The residue was dissolved in 1 mL MeOH and filtered through a 0.2 μm PTFE filter (Chromafil, Macherey-Nagel). Extracts were analyzed using HPLC-HRMS as described above.

For the isolation and characterization of sphingofungins C_3_ (5) and C_4_ (6), cultures of A. fumigatus *xyl-G sphA^Pv^* for compound extraction were prepared by inoculation of 6 preculture in Darken media (32) with 5 × 10^6^ spores/mL, incubated at 37°C and 180 rpm overnight. Also, 10 mL of these cultures were used to inoculate each 6 × 200 mL V8-based production medium (167 mL/L V8-vegetable juice; 2.2 g/L trisodium citrate dihydrate; adjusted to pH 7.0 with sodium hydroxide, 20 g/L xylose). Production cultures were incubated at 28°C for 6 days under static conditions. Extractions of production cultures were performed by addition of MeOH to a final concentration of 33% (vol/vol) to the culture followed by homogenization of the culture using an Ultra turrax T18 digital (IKA). Homogenized cultures were incubated at room temperature for 1 h and then filtered through MN 615 filter papers (Macherey-Nagel). Crude extracts were prepurified using 2,000 mg Chromabond C18ec Silica Gel columns (Macherey-Nagel). Columns were conditioned using 20 mL 100% MeOH and 20 mL water, after which the crude extract was applied to the column. The columns were first washed with water and 50% MeOH. Elution of compounds was achieved in several steps, first 70%, then 80%, then 90%, and finally 100% MeOH, each fraction was collected separately. The elution phases were then evaporated using a Genevac EZ-2 sample concentrator (SP Scientific), the residue was dissolved in up to 4 mL MeOH and filtered through a 0.2 μm PTFE filter (Chromafil, Macherey-Nagel). Individual elution steps were analyzed via HPLC-HRMS and the 80% elution phase was further purified by semipreparative HPLC using a Shimadzu LC-20AD instrument on a Kinetex C18 column (10 × 250 mm, 5 μm, 100 Å, Phenomenex) with conditions as follow: solvents A (water, 0.1% [vol/vol] formic acid) and B (acetonitrile); 10% B for 1.5 min; to 95% B over 30.5 min; 95% B for 8 min; to 10% B over 3 min; 10% B for 10 min. Fractions of HPLC runs were collected and analyzed via HPLC-HRMS. Fraction of sufficient purity of sphingofungin C_3_ or C_4_ were pooled an evaporated. Pure compound extracts were then used for NMR spectroscopy.

### NMR spectroscopy.

NMR measurements were performed on a Bruker AVANCE III 600 MHz spectrometer, equipped with a Bruker Cryoplatform. Chemical shifts are reported in parts per million (ppm) relative to the solvent residual peak (methanol-*d_4_*: ^1^H: *δ* = 3.31 ppm; ^13^C: *δ* = 49.01 ppm; CDCl_3_: ^1^H: 7.26 ppm; ^13^C: 77.16 ppm). For multiplicities of resonance signals, the following abbreviations are used: s (singlet), d (doublet), t (triplet), q (quartet), and m (multiplet) as well as combinations of these.

### Sphingofungin derivatives acquired through chemical synthesis.

2-epi sphingofungin C (7), 14-oxo sphingofungin C (8), and 14-desoxy sphingofungin C (9) were acquired from stocks synthesized previously. For synthesis and NMR analyses, see Raguž et al. (18).

### Cloning of expression constructs.

The *fadD* sequence (UniProt: P69451) was amplified from E. coli *DH5α* genomic DNA using primers 1 and 2. This was achieved by using live E. coli cells in the PCR and setting the initial 95°C boiling step to a duration of 3 min. Then the acquired DNA fragment of *fadD* was cloned into pJet1.2 using the CloneJET PCR Cloning Kit (Thermo Fisher Scientific) and sequenced, using primers 3 and 4. Afterwards, it was amplified using primers 5 and 6 and ligated into pET28a_H6TEV (33) to obtain the final pET28a_fadD plasmid. The SPT DNA sequence from Sphingomonas paucimobilis (UniProt: Q93UV0) was synthesized (Biomatik, Canada), including restriction sites for BamHI and HindIII, digested with the two restriction enzymes, and ligated into BamHI/HindIII digested pET28a_H6TEV to obtain the final pET28a_SPT plasmid.

### Expression and purification of recombinant enzymes.

FadD and SPT were produced in E. coli BL21(DE3) cells that were transformed with the respective plasmids, and grown overnight in LB media containing 25 μg/mL kanamycin at 37°C and 180 rpm. These precultures were used to inoculate a main culture containing autoinduction media (34) and 25 μg/mL kanamycin in a 1:100 ratio using baffled shaker flasks. The main culture was incubated at 37°C while shaking until an OD_600_ of ca. 1.0 was reached. Afterwards, the temperature was reduced to 18°C and the cultures were incubated overnight while shaking. Cells were harvested by centrifugation (3,000 × *g*, 4°C, 15 min) and stored at −20°C.

Bacterial cells were thawed, resuspended in buffer A (0.1 M TRIS, 0.5 M NaCl, pH 8.0), and lysed using sonication (Sonopuls 2070, Bandelin, cycle 6, 75% intensity, 2 × 2 min) on ice. The protein preparations were centrifuged (4°C, 16,000 × *g*, 15 min) and the supernatants were applied to a HisTrap FF crude column connected to an Aekta FPLC system (both GE Healthcare). After washing with 25 mM imidazole, the proteins were eluted with 500 mM imidazole using buffer B (0.1 M TRIS, 0.5 M NaCl, 0.5 M imidazole, pH 8.0). Protein containing fractions were analyzed by Coomassie-stained SDS page (Fig. S7) and pooled. FadD enzyme was stored at 4°C. For SPT, the buffer was exchanged to buffer C (20 mM TRIS, 150 mM NaCl, 10% glycerol [vol/vol], pH 8.0) using NAP-25 columns (GE Healthcare). After, it was flash frozen in liquid nitrogen and stored at −80°C. Protein concentrations were determined using Bradford assay.

### SPT assay—CoA recycling.

A step by step protocol can be found in the supplemental material.

Initially, samples containing 0.1 M HEPES pH 7.5, 2.5 mM MgCl_2_, 0.5 mM TCEP, 0.5 mM ATP, 20 μM CoA, 0.5 mM palmitic acid (50 mM stock solution in DMSO, final DMSO concentration 1% [vol/vol]), 5 mM serine, 20 μg/mL FadD, and 60 μg/mL SPT were incubated at 37°C for different time points. For extraction of 3-ketodihydrosphingosine, to confirm its formation, an equal volume of methanol was added after 2 h. The sample was filtered through a 0.2 μm PTFE filter (Chromafil, Macherey-Nagel) and analyzed by HPLC-HRMS. In all other cases, the reaction was stopped by adding ammonium molybdate (see PPi assay). Later, samples with a final DMSO concentration of 10% (vol/vol) were set up. For samples containing inhibitors (dissolved in DMSO), the final DMSO concentration was also kept at 10% (vol/vol). For blank samples, CoA was replaced with water and for samples measuring FadD activity alone SPT was replaced with buffer C. For measurements with different serine concentrations, appropriate stock solutions were used to set up the reactions. The PPi assay was performed as described before (23). All measurements were done including technical and biological triplicates.

### SPT assay—free CoA detection.

The assay was adapted from Raman et al. (16). Reactions contained 50 mM potassium phosphate pH 7.5, 0.2 mM DNTB (5,5′-Dithiobis [2-nitrobenzoic acid]), 0.25 mM palmitoyl-CoA, 60 μg/mL SPT and inhibitors in DMSO (whereas final DMSO concentration was kept at 10% vol/vol in all reactions). Reactions were started by the addition of serine to a concentration of 5 mM. Blanks did not contain serine and control reactions did not contain any inhibitors, but DMSO concentration was kept at 10%. Assays were performed with 0.1 mL reactions in 96-well plates (BRANDplates, VWR, Darmstadt) at 37°C and measured in 3-min intervals at 412 nm in a CLARIOstar plate reader (BMG Labtech, Ortenberg, Germany).

### FadD assay.

The reaction mix, containing 20 mM HEPES pH 7.5, 2.5 mM MgCl_2_, 0.5 mM TCEP, 0.5 mM ATP, 0.5 mM palmitic acid (50 mM stock solution in DMSO), 0.5 mM CoA, 20 μg/mL FadD, and inhibitors dissolved in DMSO (whereas final DMSO concentration was kept at 10% in all reactions), was quickly vortexed and incubated at 37°C for 3 min. The reaction was stopped by adding ammonium molybdate (see PPi assay). For blank samples, CoA was replaced with water. For negative controls, the inhibitors were replaced with DMSO. The PPi assay was performed as described before (23). For inhibitors, 1 μM myriocin or 15 μM sphingofungin B (1), C (2), C_3_ (5), or C_4_ (6) were used. All measurements were done including technical and biological triplicates.

### Yeast inhibitory assay.

Growth inhibition of S. cerevisiae (strain BY4741) by SIs (myriocin, 2-epi sphingofungin C, 14-oxo sphingofungin C,14-desoxy sphingofungin C, sphingofungins B, C, B_2_, C_2_, C_3_ and C_4_) was determined by measuring the reduction of resazurin to the fluorescent resorufin (29). The reduction was determined by measuring fluorescence using a CLARIOstar plate reader (BMG Labtech, Ortenberg, Germany) with an excitation of 570 nm and measuring emission at 615 nm. Assays were conducted in 96-well plates (BRANDplates, VWR, Darmstadt) with 150 μL samples at 30°C, measuring fluorescence every 30 min for 13 h. Samples consisted of SD media containing 1% (vol/vol, final concentration) of DMSO, 0.002% (wt/vol, final concentration) resazurin, and S. cerevisiae cells at a final OD_600_ of 0.01 (S. cerevisiae cells were inoculated from an SD overnight culture at 180 rpm and 30°C). Blank controls did not contain any cells. Samples contained different concentrations of the SIs ranging from 0.3125 to 20 μM for myriocin (Sigma-Aldrich; Merck KGaA, Darmstadt, Germany), sphingofungin B and B_2_, and 1.25 to 40 μM for 2-epi sphingofungin C, 14-oxo sphingofungin C,14-desoxy sphingofungin C, sphingofungin C and C_2_, and 0.625 to 40 μM for sphingofungin C_3_ and C_4_. Samples containing no SI’s were used as negative controls and samples supplemented with hygromycin B (Invivogen, 200 μg/mL final concentration), instead of SIs, were used as positive controls to determine inhibition of growth. All presented results have been obtained from measurements performed in technical and biological triplicates. Relative inhibition was calculated using the following formula:

$$\frac{\text{fluorescence of sample at }13\text{ h }-\text{ fluorescence of sample at }0\text{ h }}{\text{fluorescence of negative control at }13\text{ h }-\text{ fluorescence of negative control at }0\text{ h}}$$

Samples with a relative inhibition below the relative inhibition threshold determined by the positive control (hygromycin B) were considered inhibiting, whereas samples significantly above the threshold were considered noninhibiting.

Approximate IC_50_ values were calculated using the “GraphPad Prism 9.3.1” software using its nonlinear regression functions (Table S3).
