# Supplementary material for: Desaturation of the Sphingofungin Polyketide Tail Results in Increased Serine Palmitoyltransferase Inhibition
Source: Microbiol Spectr. 2022 Sep 19;10(5):e01331-22. doi: 10.1128/spectrum.01331-22 (PMC9603476; doi:10.1128/spectrum.01331-22)
Supplement: Supplemental file 1 — Supplemental material. Download spectrum.01331-22-s0001.pdf, PDF file, 1.2 MB [file spectrum.01331-22-s0001.pdf]

Supporting Information

Desaturation of the sphingofungin polyketide tail results in increased serine palmitoyltransferase inhibition

Sandra Hoefgen‡[1], Alexander U. Bissell‡[1,2], Ying Huang[1,2], Fabio Gherlone[1,2], Luka Raguž[3,4], Christine Beemelmanns[3] and Vito Valiante[1]#

[1]Biobricks of Microbial Natural Product Syntheses, Leibniz Institute for Natural Product Research and Infection Biology, Hans Knöll Institute (HKI), Jena, Germany

[2]Faculty of Biological Sciences, Friedrich Schiller University Jena, Germany

[3]Chemical Biology of Microbe-Host Interactions, Leibniz Institute for Natural Product Research and Infection Biology, Hans Knöll Institute (HKI), Jena, Germany

[4]Faculty of Chemistry and Earth Sciences, Friedrich Schiller University Jena, Germany

*Corresponding Author: E-mail: vito.valiante@leibniz-hki.de

SPT assay - Step by Step protocol

Ammoniummolybdate solution: 25 mg/ml in 5N H_2SO_4 (freshly prepared and stored on ice)

Fiske-Subbarow Reducer: 148 mg/ml in H_2O (freshly prepared)

β-mercaptoethanol: 0.5 M in H_2O (freshly prepared)

SPT reaction mix:

**Chemical**	**Stock solution**	**Final concentration**
HEPES pH 7.5	1.0 M	0.1 M
$MgCl_2$	50 mM	2.5 mM
TCEP	10 mM	0.5 mM
ATP	5 mM	0.5 mM
CoA	1 mM	20 μM[a]
Palmitic acid (DMSO)	50 mM	0.5 mM
Serine	50 mM	5 mM
Inhibitor (DMSO)	5 mM	varying
DMSO		10%[b] (V/V)
FadD	0.6 mg/ml	20 µg/ml
SPT	2.9 mg/ml	60 µg/ml

[a]For the blank CoA was replaced with water

[b]Keep final DMSO concentration at 10% (including the volume of palmitic acid and inhibitor you add). If less than 10% DMSO then add up to 10%

Step by Step

- ➔ 1). Pipet together (HEPES pH 7.5, $MgCl_2$, TCEP, ATP, CoA, Serine, Inhibitor (DMSO), DMSO), add palmitic acid last and mix.
- ➔ 2). Add enzymes and mix again carefully.
- ➔ 3). Incubate at 37 °C (for endpoint measurements for 2h).
- ➔ 4). Add 14 µl Ammoniummolybdate solution per 100 µl sample and mix well (the reaction is stopped).
- ➔ 5). Transfer 114 µl to a 96-well plate.
- ➔ 6). Add 6 µl Fiske-Subbarow-Reducer and mix well using a pipet
- ➔ 7). Add 14 µl β-mercaptoethanol and mix well using a pipet
- ➔ 8). Incubate at room temperature for 10 min and measure absorption at 580 nm

Experimental

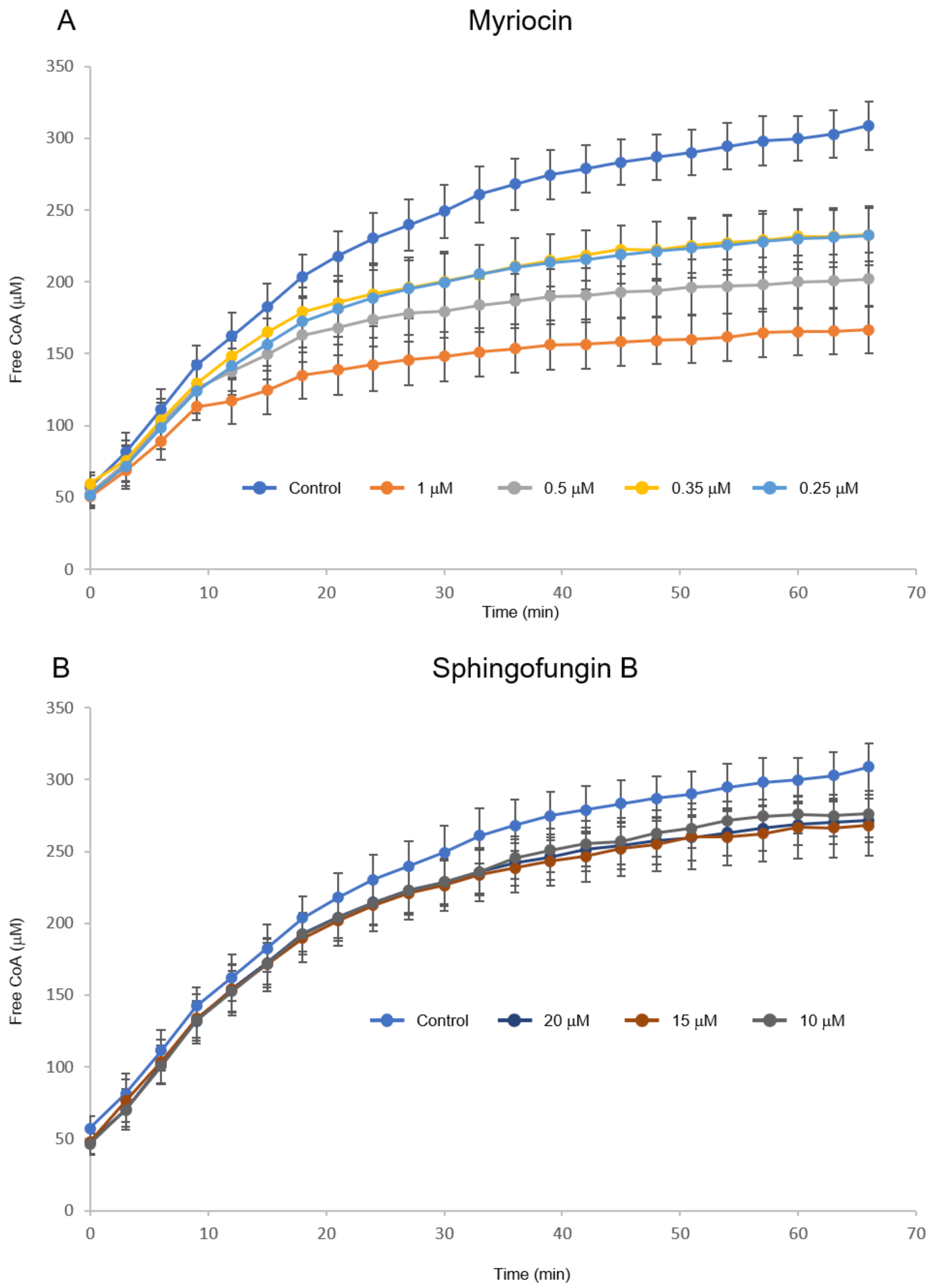

FIG S1. SPT activity assays by free CoA detection. SPT activity shown as production of free CoA over time in the presence of increasing concentrations (as shown in the figure) of the SPT inhibitors (**A**) myriocin and (**B**) sphingofungin B. All experiments were conducted in technical and biological triplicates. Error bars represent standard errors.

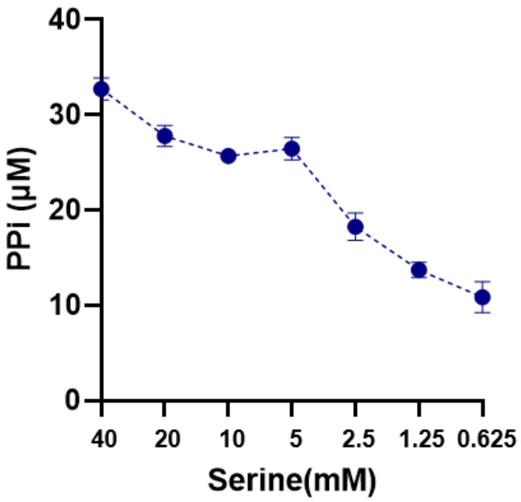

Fig S2. SPT Assay with varying serine concentrations. Shown is the SPT activity in PPi (µM), as measured with different concentrations of serine (as indicated in the figure). All experiments were conducted in technical and biological triplicates, shown error bars represent the calculated standard errors.

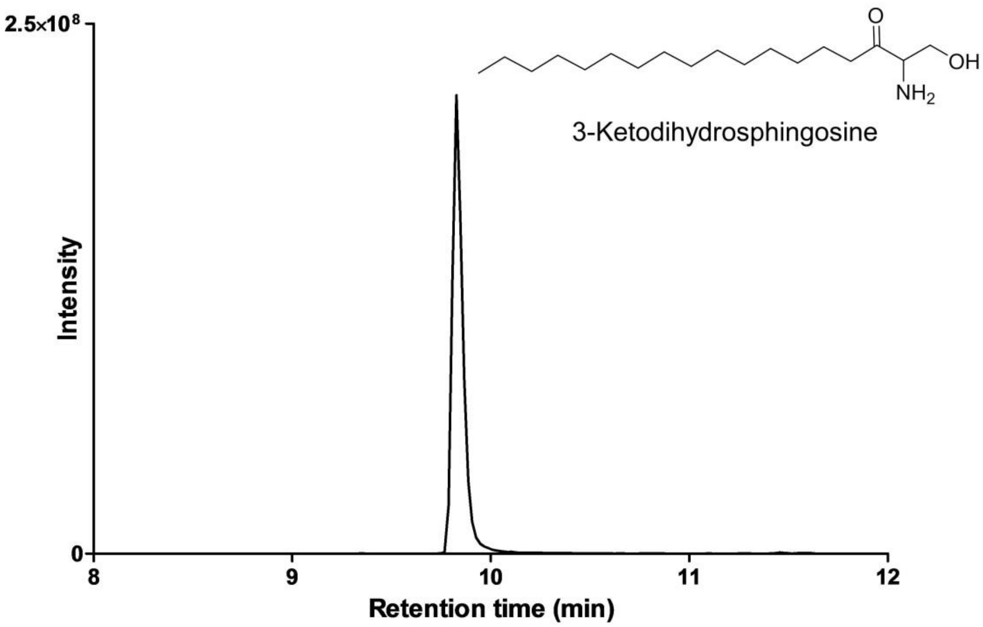

FIG S3. HPLC-HRMS analysis of 3-ketodihydrosphingosine. The extracted ion chromatogram for m/z = 300.2897 ± 5 ppm, corresponding to $[M+H]^+$ of 3-ketodihydrosphingosine. The compound has been extracted from the performed *in vitro* SPT assay reported in Figure 2 of the main article.

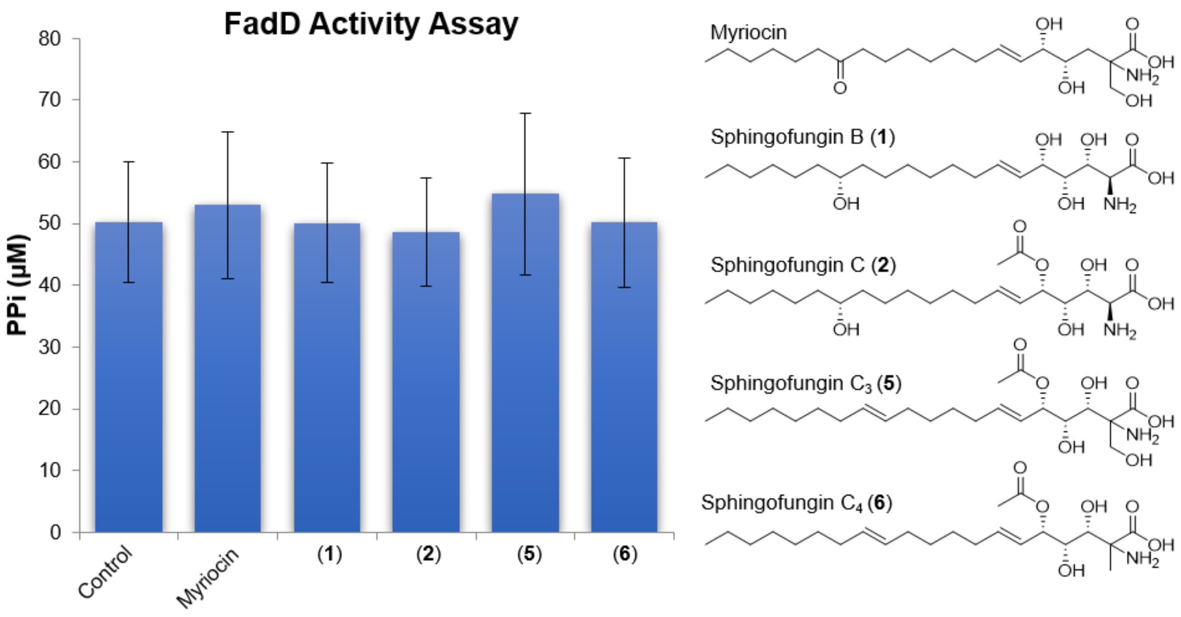

FIG S4. FadD activity assay. Shown are the result of an activity assay with FadD, performed to investigate the influence of different SIs on the activity of the enzyme. Tested was myriocin at 1 µM concentration and the different sphingofungin derivatives B (**1**), C (**2**), B_2 (**3**) and C_2 (**4**), at concentrations of 15 µM. The control did not contain any inhibitor. Also shown are the chemical structures of each compound. All experiments were conducted in technical and biological triplicates. Error bars show standard errors.

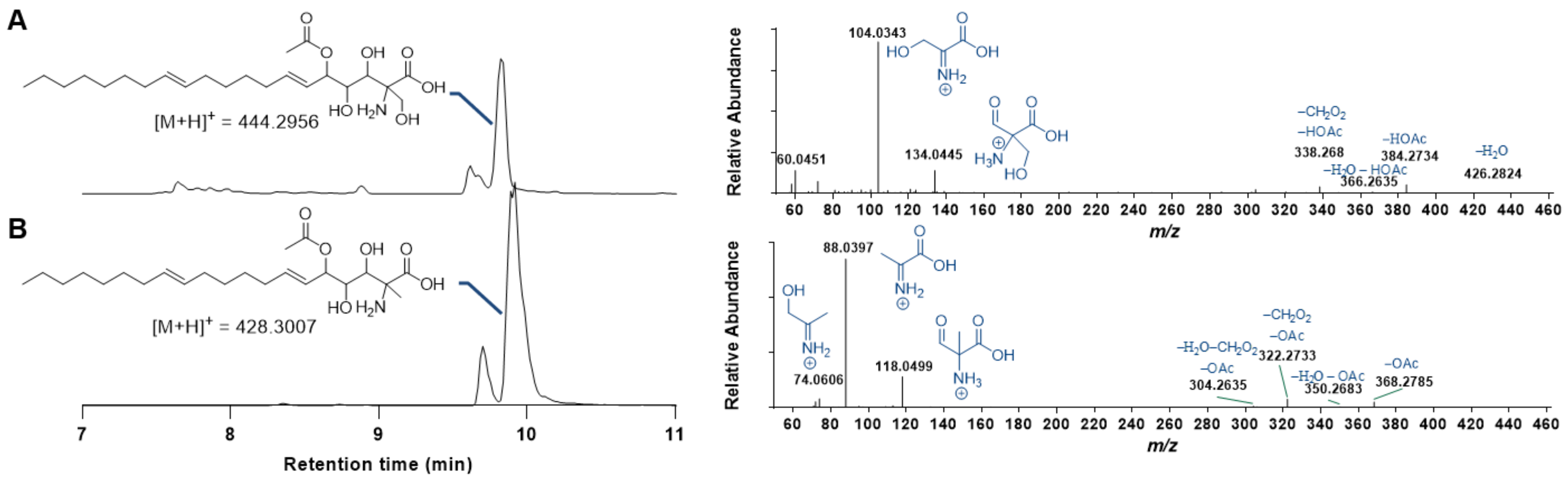

FIG S5. Sphingofungin pathway intermediates identified in xyl-G sphABs strain. Shown are EICs of HPLC-HRMS analysis in conjunction with obtained HRMS[2] spectra of marked peaks, as well as predicted structures of compounds. Assignable ions or neutral losses are displayed in blue. **A**, Sphingofungin C3 (**5**) with $[M+H]^+$ 444.2956 and HRMS[2] spectra. **B**, Compound Sphingofungin C4 (**6**) with $[M+H]^+$ 428.3007 and HRMS[2] spectra.

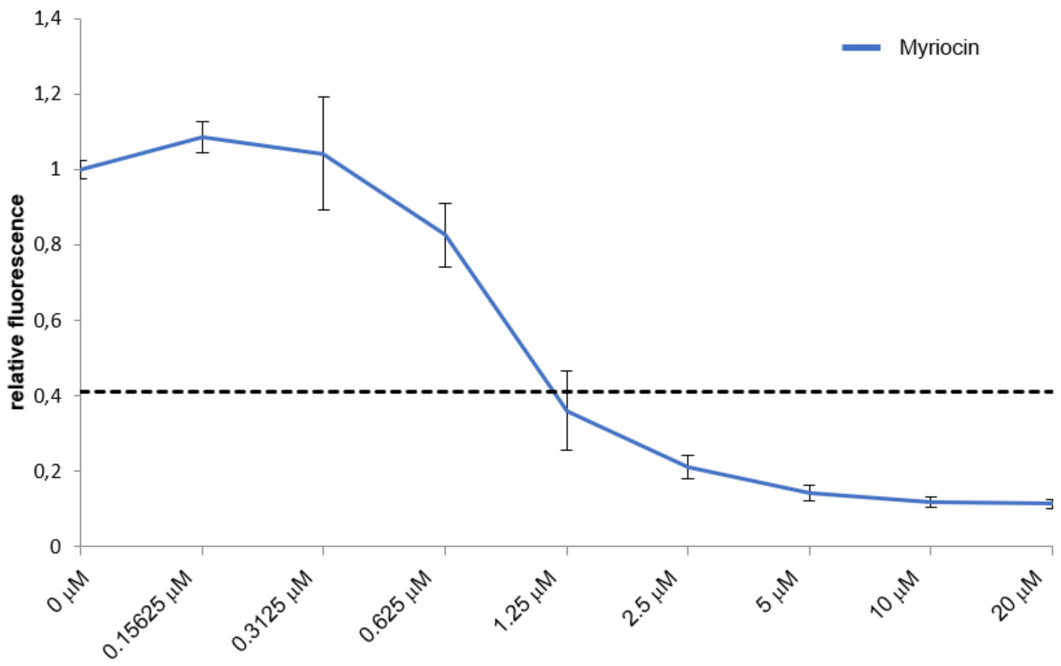

Fig S6. *In vivo* yeast resazurin assay with myriocin. Metabolic activity is represented as relative fluorescence (y-axis) depending on the concentration of the used inhibitor (x-axis). The black dotted line indicates the relative fluorescence observed at an inhibiting concentration of hygromycin B and functions as the threshold at which *S. cerevisiae* is considered fully inhibited. Myriocin concentrations were used as indicated by the figure. All experiments were conducted in technical and biological triplicates, shown error bars represent the calculated standard errors.

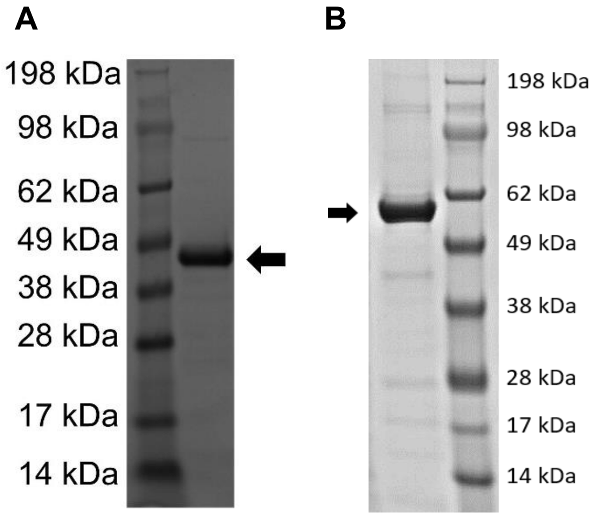

FIG S7. SDS page of purified enzymes. 2 µg of (**A**) SPT (theoretical MW: 48 kDa) and (**B**) FadD (theoretical molecular weight (MW): 65 kDa), were applied to a Coomassie stained SDS-PAGE. Bands of the corresponding proteins are marked with an arrow.

Table S1. ^{1}H (600 MHz) and ^{13}C (150 MHz) NMR data for Sphingofungin C$_3$ (**5**) in methanol-d_4

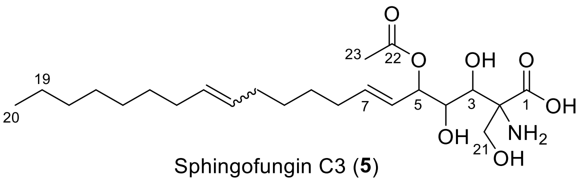

Sphingofungin C3 (**5**)

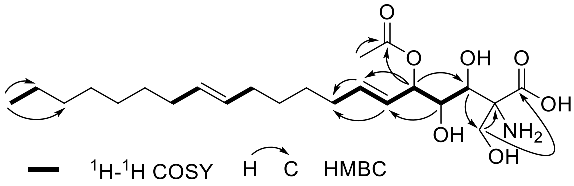

Position	δ_C	δ_H, mult (J in Hz)
**1**	173.0	
**2**	71.1	
**3**	68.7	3.94, d (1.1)
**4**	74.9	3.88, dd (8.6, 1.1)
**5**	77.9	5.35, overlap
**6**	125.7	5.37, overlap
**7**	139.1	5.87, m
**8**	33.3	2.06, overlap
**9**	30.8[a]	1.25-1.44, overlap
**10**	30.3[a]	1.25-1.44, overlap
**11**	30.3[a]	1.25-1.44, overlap
**12**	30.2[a]	1.25-1.44, overlap
**13**	33.6	1.94-2.01, overlap
**14**	131.7	5.39, overlap
**15**	131.3	5.39, overlap
**16**	33.4	1.94-2.01, overlap
**17**	29.3[a]	1.25-1.44, overlap
**18**	33.0	1.28, overlap
**19**	23.7	1.31, overlap
**20**	14.4	0.90, t (7.0)
**21**	65.0	3.98, d (11.0)
		3.84, d (11.0)
**22**	172.4	
**23**	21.3	2.04, s

[a] Values are interchangeable.

Table S2. ^{1}H (600 MHz) and ^{13}C (150 MHz) NMR data for sphingofungin C4 (**6**) in methanol-d_4

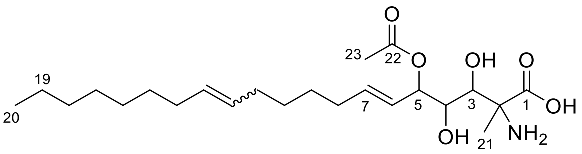

Sphingofungin C4 (**6**)

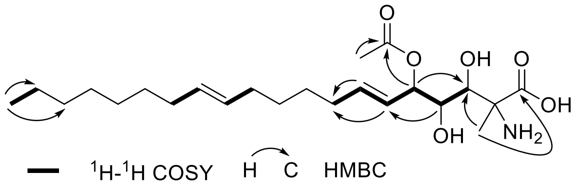

Position	δ_C	δ_H, mult (J in Hz)
**1**	175.2	
**2**	66.7	
**3**	71.2	3.85, d (1.1)
**4**	74.8	3.92, dd (8.6, 1.1)
**5**	78.2	5.36, overlap
**6**	125.7	5.38, overlap
**7**	139.1	5.87, m
**8**	33.0	2.06, overlap
**9**	30.8[a]	1.25-1.43, overlap
**10**	30.3[a]	1.25-1.43, overlap
**11**	30.3[a]	1.25-1.43, overlap
**12**	30.2[a]	1.25-1.43, overlap
**13**	33.6	1.95-2.00, overlap
**14**	131.6	5.39, overlap
**15**	131.3	5.39, overlap
**16**	33.4	1.95-2.00, overlap
**17**	29.3[a]	1.25-1.43, overlap
**18**	33.0	1.28, overlap
**19**	23.7	1.29, overlap
**20**	14.4	0.90, t (7.0)
**21**	21.9	1.50, s
**22**	172.6	
**23**	21.3	2.04, s

[a] Values are interchangeable.

Table S3. Approximate IC_{50} values and fully inhibiting concentrations of SPT inhibitors used in *in vivo* resazurin assays with *S. cerevisiae*.

Compound	**Approximate IC_{50} *S. cerevisiae* [µM]**	**"Fully" inhibiting concentration against *S. cerevisiae* [µM]**
Myriocin	0,8971	1,25
Sphingofungin B (**1**)	1,019	2,5
Sphingofungin C (**2**)	5,635	20
Sphingofungin B_2 (**3**)	0,6906	2,5
Sphingofungin C_2 (**4**)	0,5738	2.5
Sphingofungin C_3 (**5**)	0,6106	2.5
Sphingofugnin C_4 (**6**)	>40	>40
2-epi sphingofungin (**7**)	>40	>40
14-oxo sphingofungin C (**8**)	8,838	20
14-desoxy sphingofungin C (**9**)	4,542	10

Table S4. Primers used in this study.

No.	Oligonucleotide	Sequence (5`- 3`)
1	pJet_fadD_fw	ATGAAGAAGGTTTGGCTTAACC
2	pJet_fadD_rv	TCAGGCTTTATTGTCCACTTTG
3	pJet_For	CTTGTGCCTGAACACCATATCC
4	pJet_Rev	CAGCCTGAAAATCTTGAGAG
5	pET28a_fadD_fw	TTTTTGGATCCAAGAAGGTTTGGCTTAACCG
6	pET28a_fadD_rv	TTTTTAAGCTTTCAGGCTTTATTGTCCACTTTG

Table S5. Plasmids used and created in this study.

Name	Relevant Feature	Reference
pJet1.2-fadD	pJET1.2, fadD	This study
pET28a-H6TEV	pET28a, H6TEV	Huber(1)
pET28a-fadD	pET28a, fadD, H6TEV	This study
pET28a-SPT	pET28a, *SPT*, H6TEV	This study

Note S1. NMR analysis of sphingofungin C$_4$

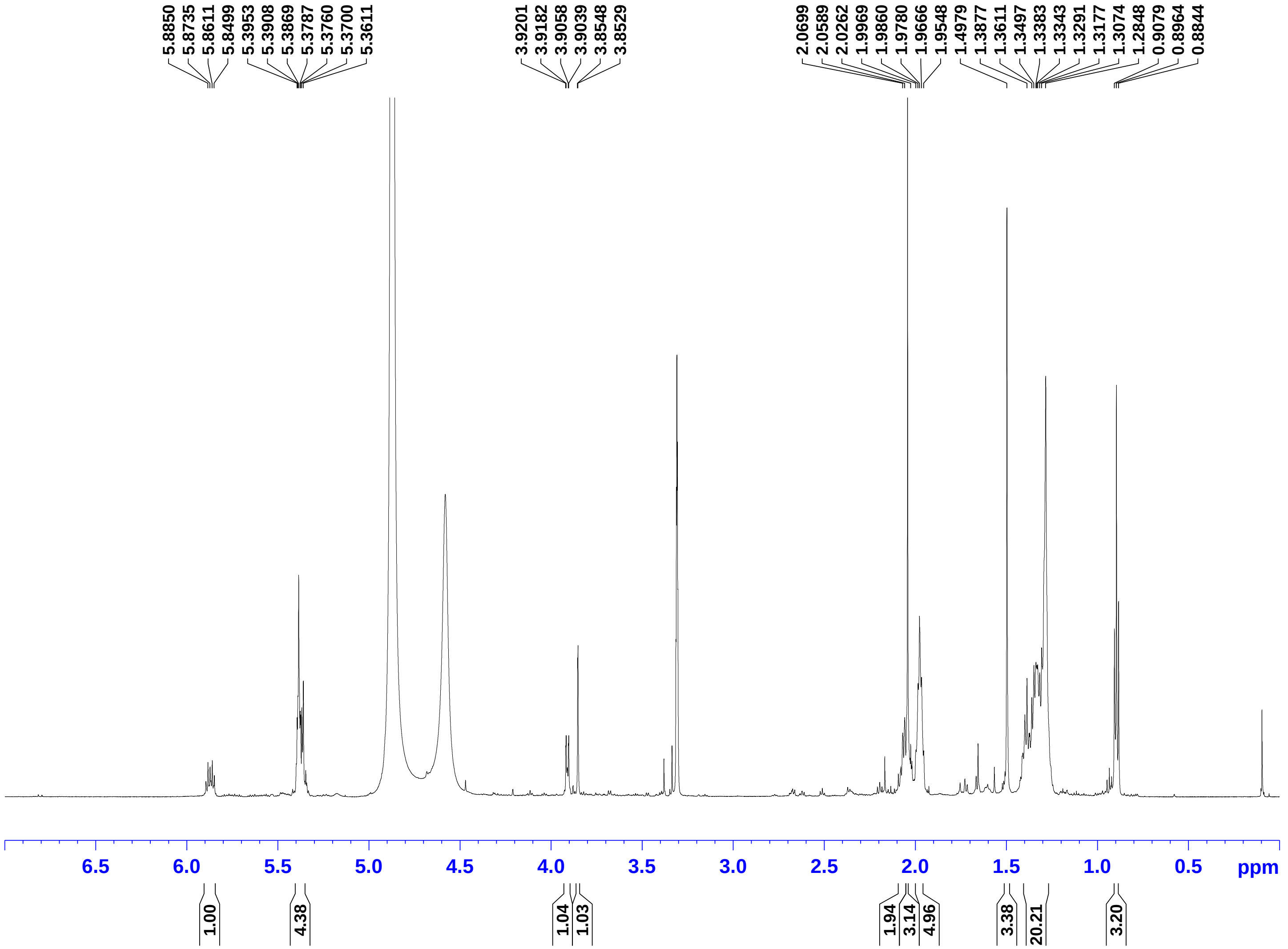

^{1}H NMR (600 MHz, methanol-d_4) spectrum of compound **sphingofungin C$_4$**

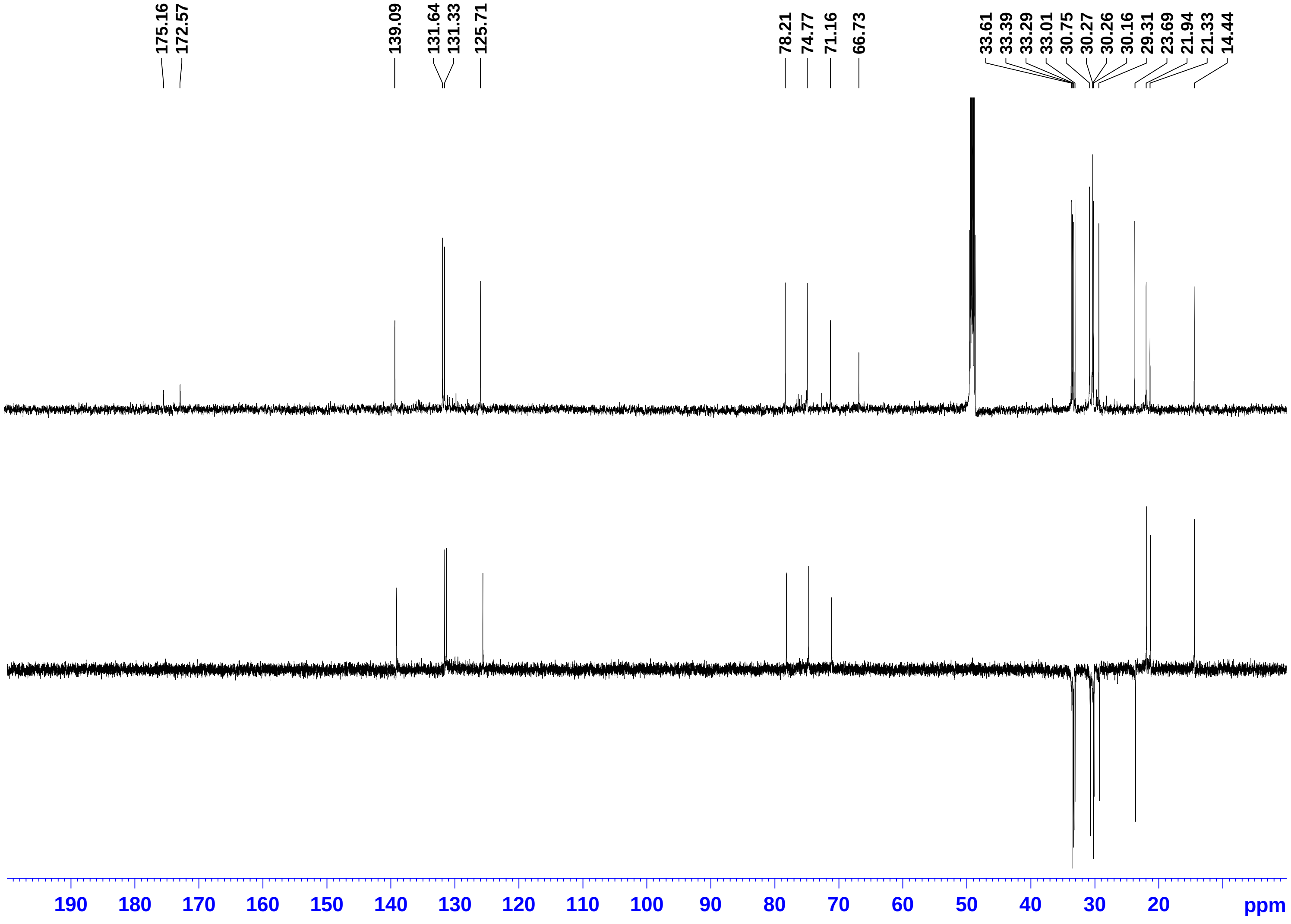

^{13}C and DEPT135 NMR (150 MHz, methanol-d_4) spectrums of compound **sphingofungin C$_4$**

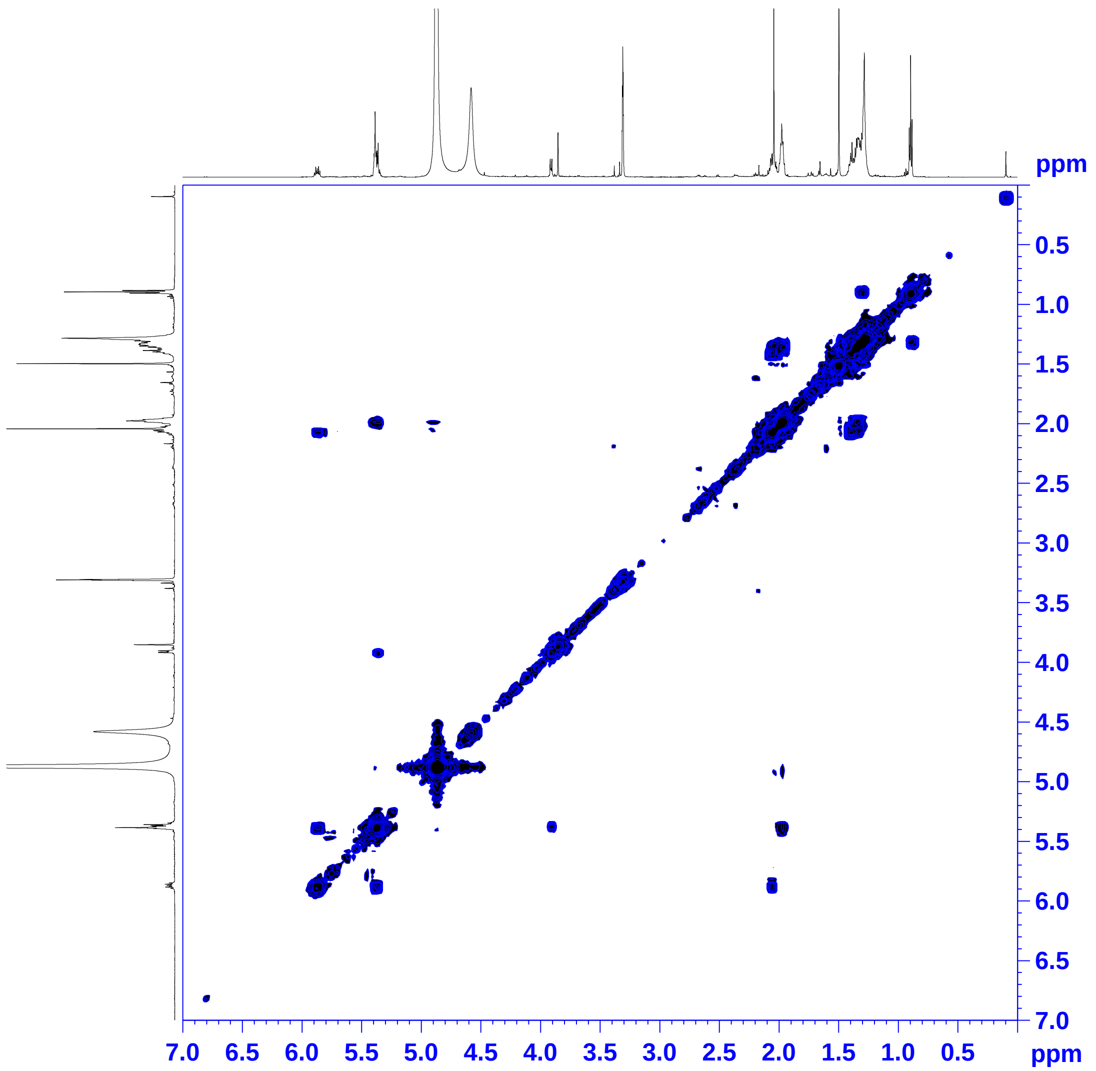

COSY spectrum of compound **sphingofungin C_4**

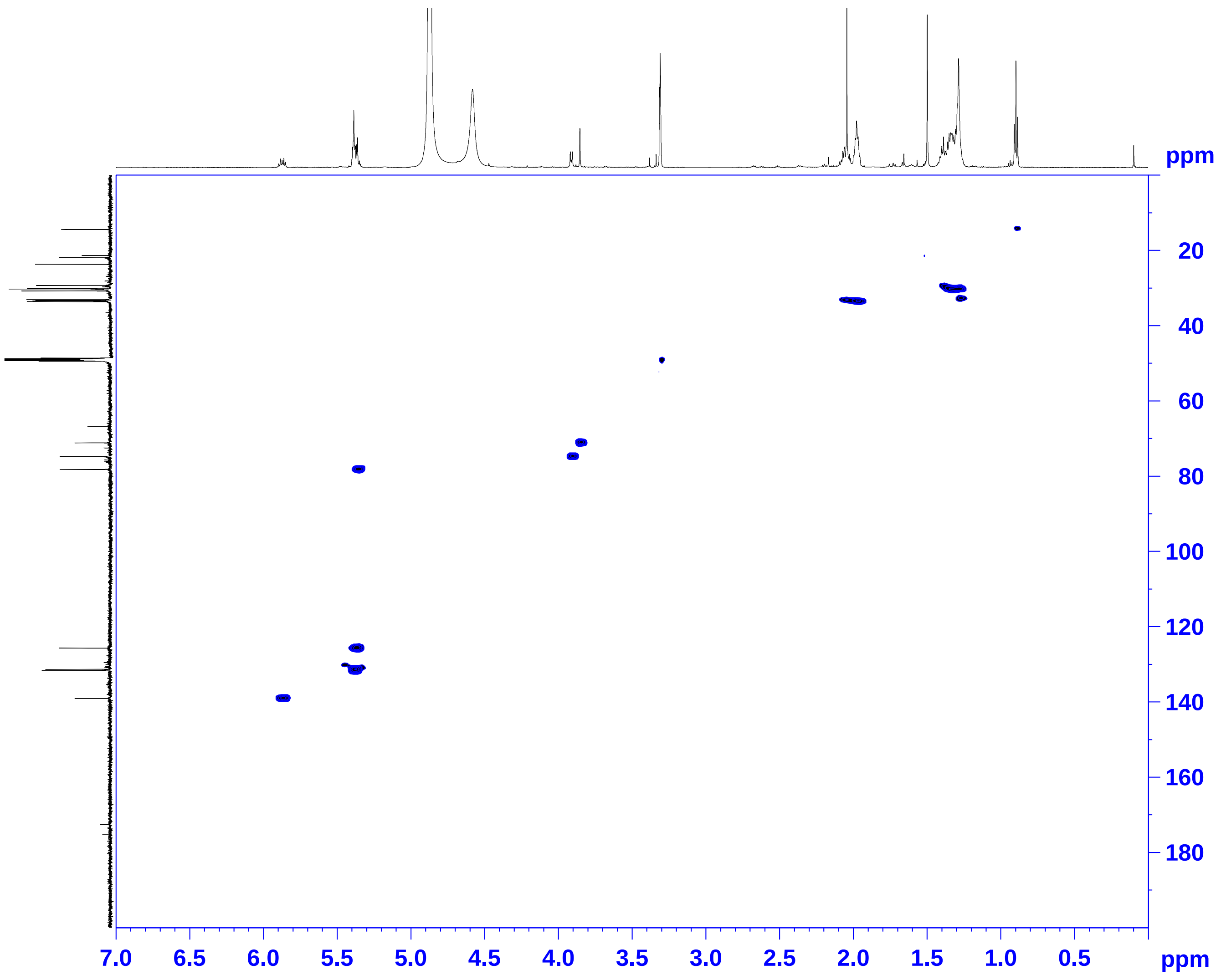

HSQC spectrum of compound **sphingofungin C_4**

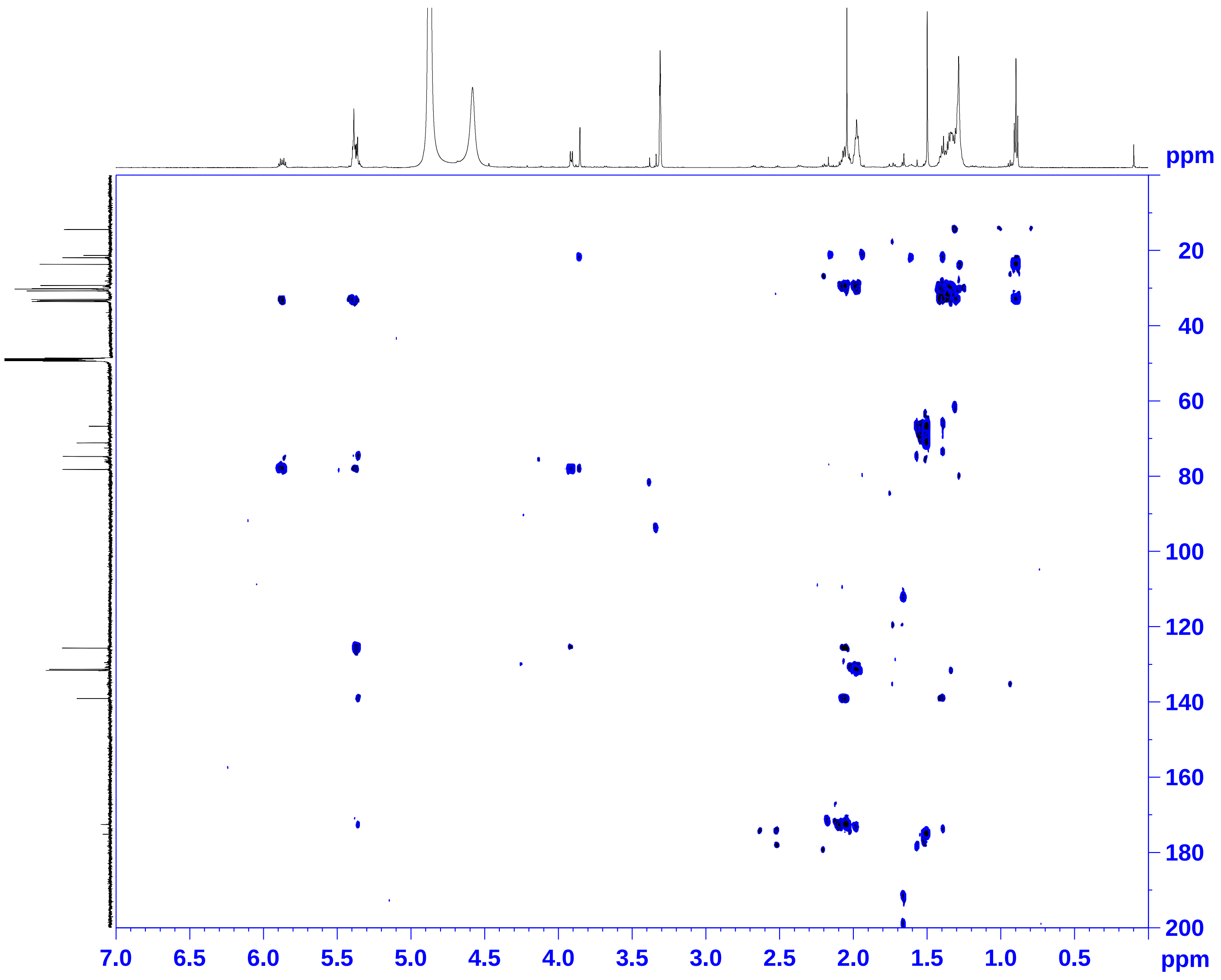

HMBC spectrum of compound **sphingofungin C_4**

Note S2. NMR analysis of sphingofungin C3

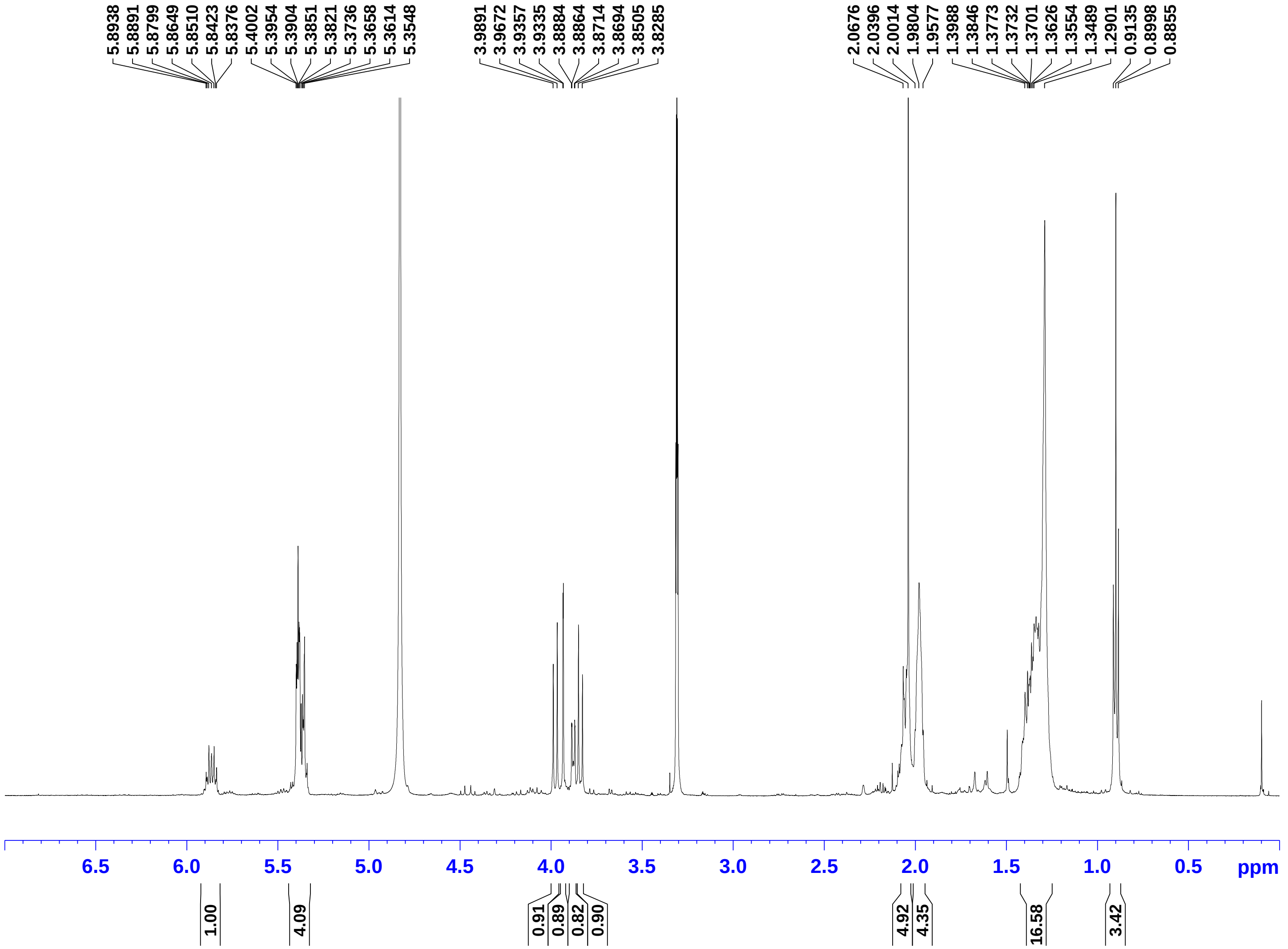

^{1}H NMR (600 MHz, methanol-d_4) spectrum of compound **sphingofungin C$_3$**

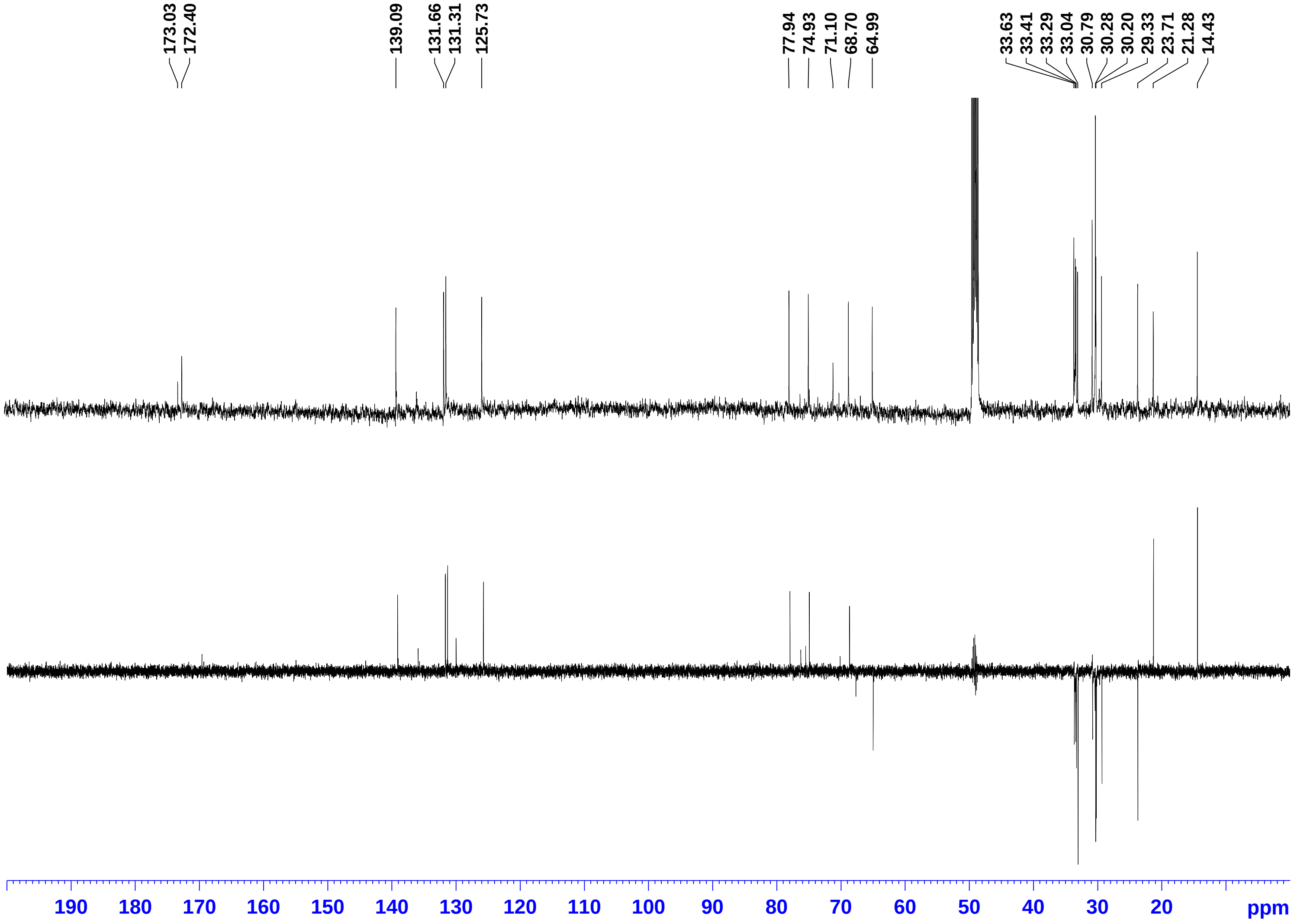

^{13}C and DEPT135 NMR (150 MHz, methanol-d_4) spectrums of compound **sphingofungin C_3**

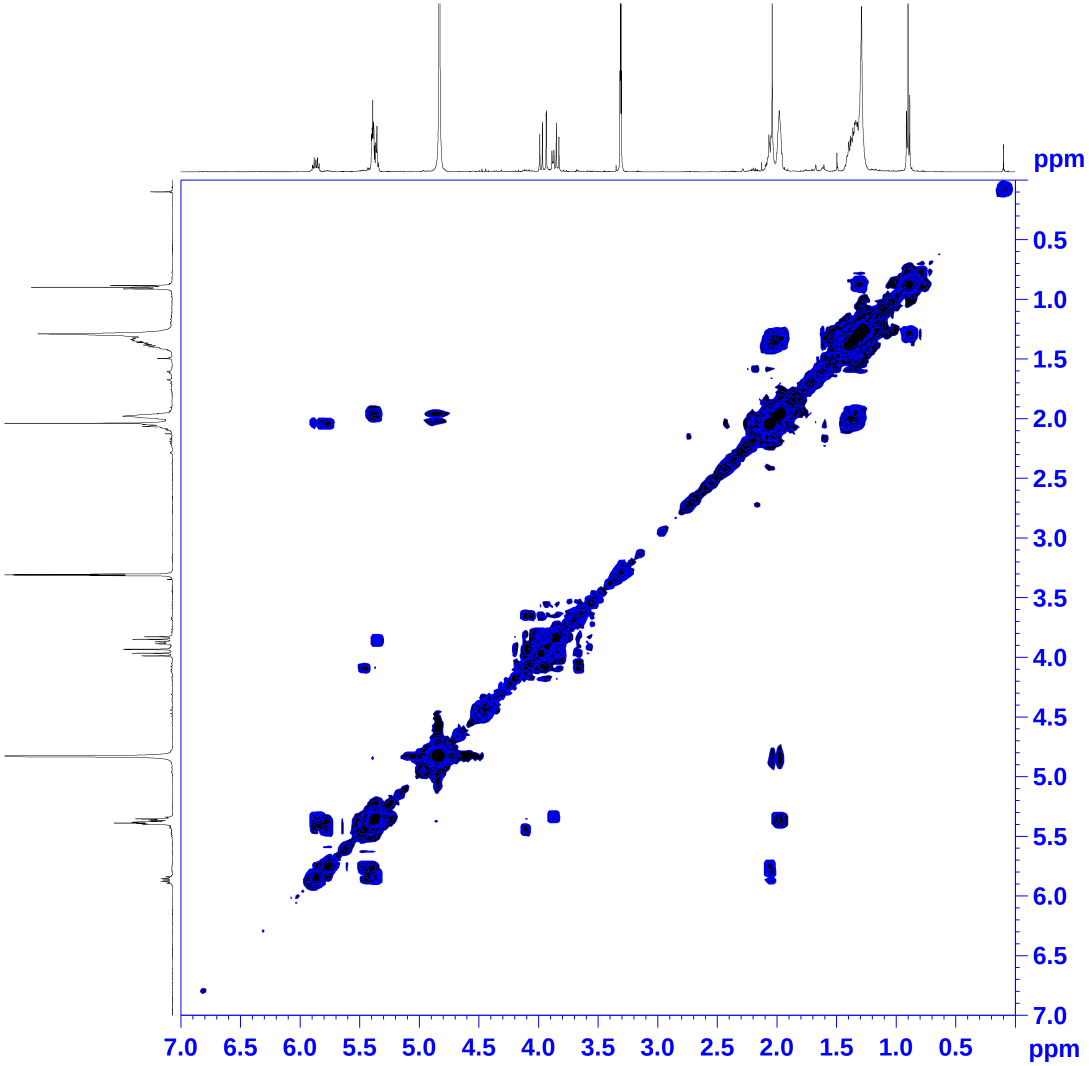

COSY spectrum of compound **sphingofungin C_3**

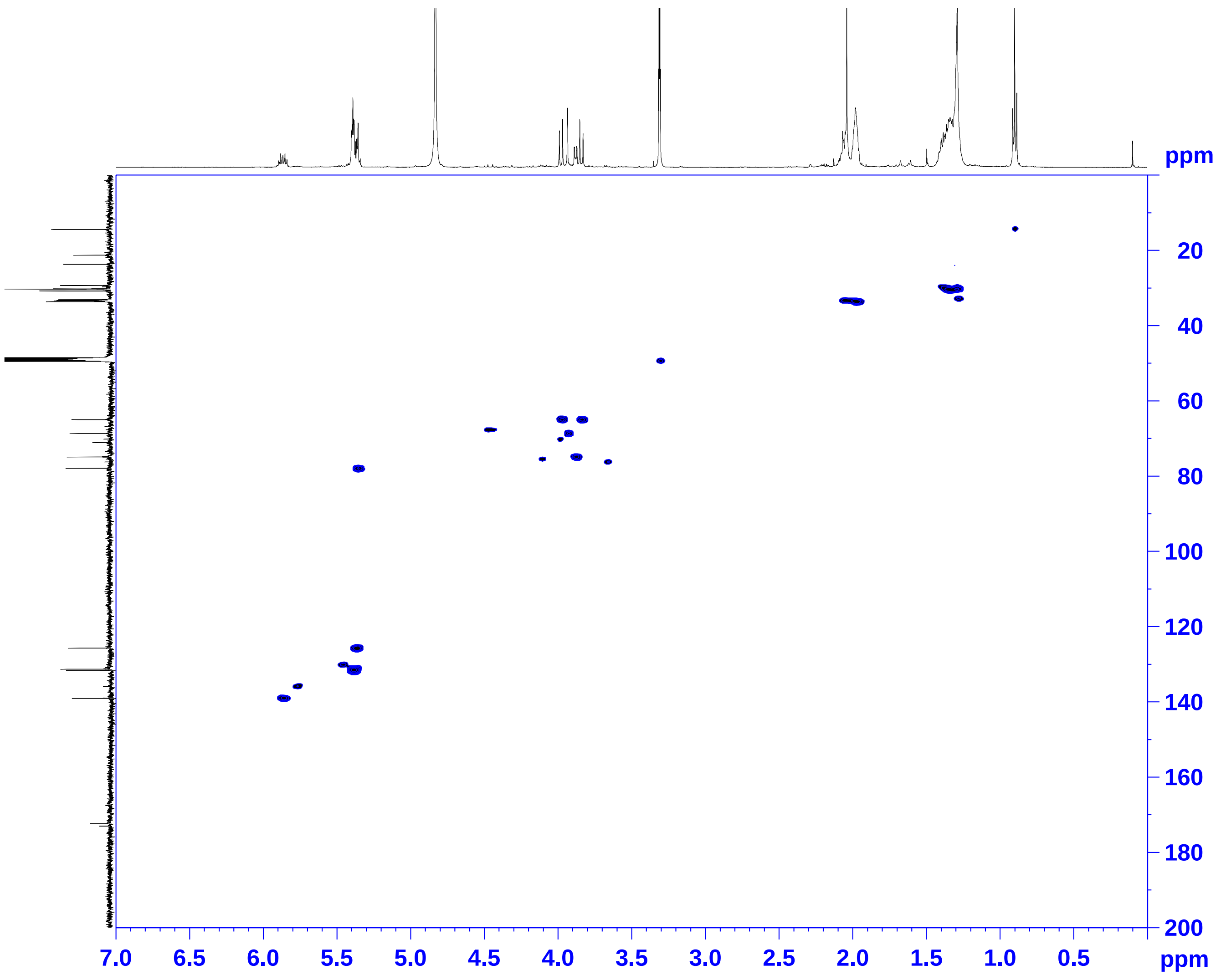

HSQC spectrum of compound **sphingofungin C_3**

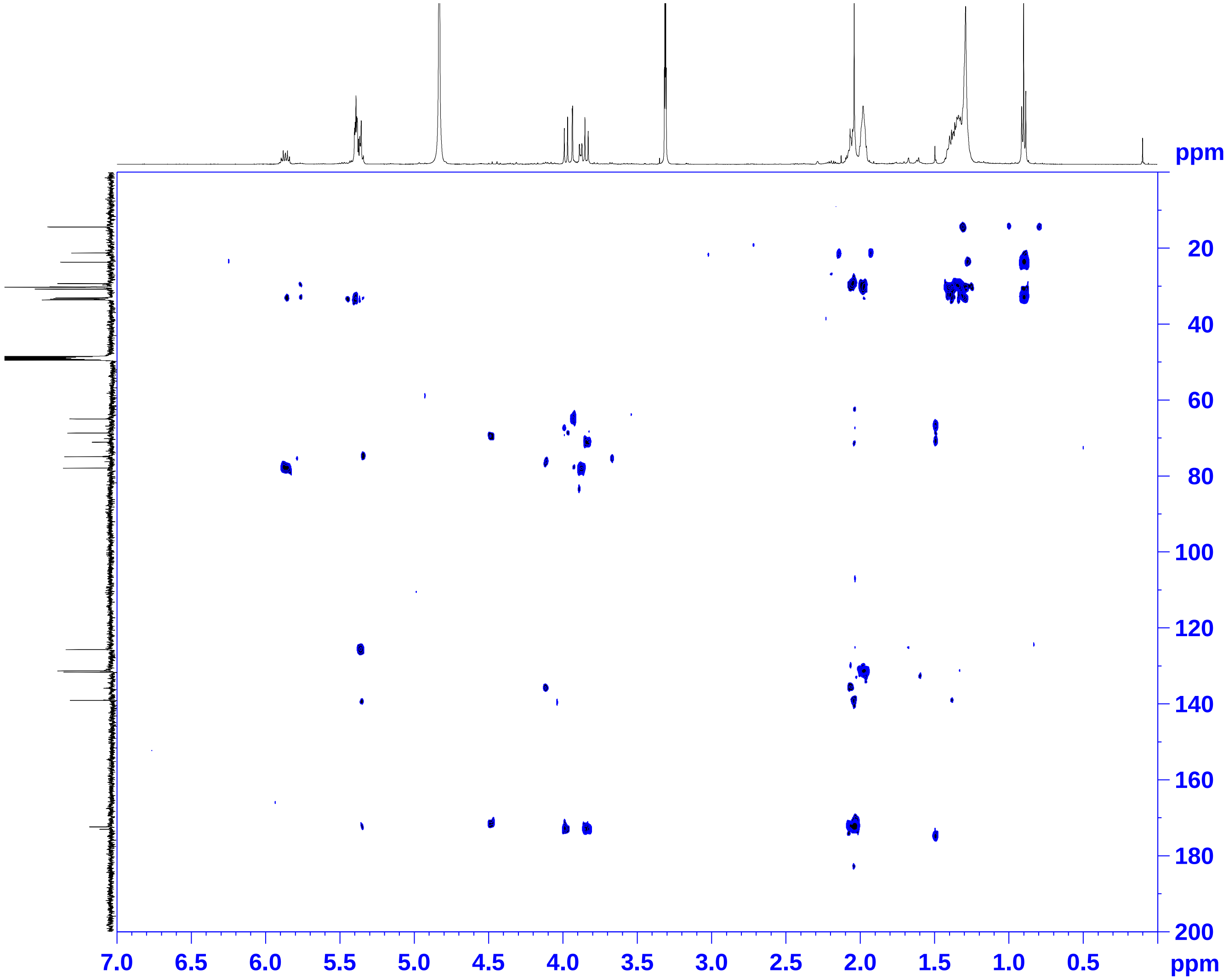

HMBC spectrum of compound **sphingofungin C_3**

References

1. Huber EM, Scharf DH, Hortschansky P, Groll M, Brakhage AA. 2012. DNA minor groove sensing and widening by the CCAAT-binding complex. Structure 20:1757-68.
